# Design of a novel tribromide ionic liquid covalent triazine framework for green synthesis of benzimidazoles

**DOI:** 10.1039/d6ra04048a

**Published:** 2026-07-03

**Authors:** Azin Kharazmi, Ramin Ghorbani-Vaghei, Ardeshir Khazaei, Idris Karakaya, Rahman Karimi-Nami

**Affiliations:** a Department of Organic Chemistry, Faculty of Chemistry and Petroleum Sciences, Bu-Ali Sina University Hamedan 6517838683 Iran rgvaghei@yahoo.com A.RGV@guilan.ac.ir +988138380709 +989183122123; b Department of Organic Chemistry, Faculty of Chemistry, University of Guilan Rasht Iran; c Department of Chemistry, College of Basic Sciences, Gebze Technical University 41400 Gebze Turkiye; d Department of Chemistry, Faculty of Science, University of Maragheh P. O. Box 55181-83111 Maragheh Iran

## Abstract

Ionic covalent triazine frameworks are a class of porous materials that integrate ionic liquid moieties into the backbone or pores of CTF. This combination imparts unique properties such as high ionic conductivity, enhanced catalytic activity, and improved affinity for polar or charged species. A novel tribromide ionic liquid covalent triazine framework catalyst was synthesized through the combination of 4,4′-(butane-1,4-diylbis(oxy))dibenzaldehyde and *N*^1^,*N*^1^′-(6-(2-aminobenzyl)-1,3,5-triazine-2,4-diyl)bis(benzene-1,2-diamine), denoted as [IL-BD-AT]^+^Br_3_^−^. Its elevated nitrogen content provides abundant anchoring sites for bromine species, thereby enhancing catalytic performance. This rational design significantly reduces the intrinsic toxicity of bromine and minimizes associated environmental hazards. Furthermore, the catalyst exhibits excellent thermal stability, ensuring robust performance under diverse reaction conditions. The catalyst demonstrated exceptional catalytic activity, achieving high yields of benzimidazole derivatives from the reaction of various aldehydes and 1,2-phenylenediamine (up to 98%) under mild conditions within short reaction times (5–20 min). Notably, the catalyst was easily recovered by centrifugation and reused for six consecutive cycles without significant loss of activity. Owing to its insolubility, the catalyst can be readily separated *via* centrifugation, thereby improving its applicability in sustainable catalytic processes. This study underscores the potential of covalent triazine frameworks in developing efficient, thermally stable, and environmentally benign catalytic systems for organic synthesis. The structural and physicochemical characteristics of the catalyst were comprehensively elucidated using a range of analytical techniques.

## Abstract 2

Ionic covalent triazine frameworks are a class of porous materials that integrate ionic liquid moieties into the backbone or pores of CTF.

## Graphical abstract

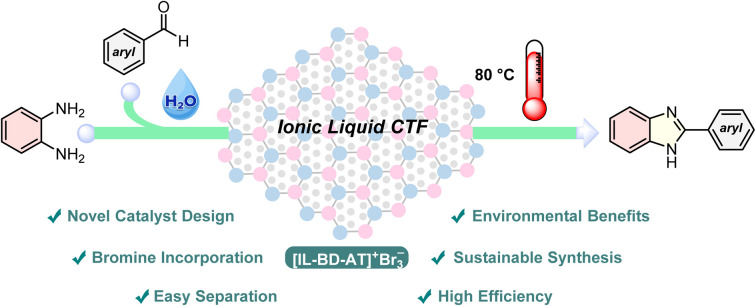

## 1. Introduction

The field of green chemistry establishes a comprehensive framework for reducing the environmental impact of chemical processes through the application of twelve fundamental principles.^1,2^ These principles address diverse aspects of chemical synthesis and manufacturing, ranging from the design of inherently safer processes to the minimization of waste generation and the promotion of renewable feedstocks.^3,4^ Among the various strategies that align with these principles, catalysis occupies a central position as a cornerstone of green chemistry.^5,6^ Catalytic methodologies enhance reaction efficiency, lower energy demands, and reduce the reliance on hazardous reagents and solvents, thereby contributing significantly to sustainable chemical development.^3,5,7^

Within this context, covalent triazine frameworks (CTFs) a subclass of porous organic polymers within the broader family of covalent organic frameworks (COFs) have emerged as promising materials for catalytic applications due to their structural tunability, chemical stability, and high surface area.^8^ These materials are distinguished by their nitrogen-rich composition, fully conjugated backbone, and exceptionally high surface area.^8^ CTFs are typically synthesized through the trimerization of nitrile-containing monomers, resulting in a robust network of triazine rings interconnected by covalent bonds.^9^ The distinctive structural features of CTFs, including their exceptional chemical stability and tunable band gaps, arise from their π–π stacking architecture.^10^ As a subclass of COFs, CTFs share several characteristics with other porous materials; however, they are distinguished by their triazine-based building units and the unique synthetic strategies employed in their construction.^11^

In recent years, growing scholarly attention has been directed toward CTFs, largely due to their multifunctional properties and their potential applicability across diverse fields of science and technology. These porous organic polymers, defined by their nitrogen-rich composition and fully conjugated backbone, possess distinctive physicochemical properties that render them highly suitable for a broad spectrum of applications.^12,13^ CTFs have emerged as promising materials for photocatalytic applications, particularly in hydrogen evolution reactions. Their tunable electronic structures, coupled with the ability to form heterojunctions with other semiconductors, contribute to significantly enhanced photocatalytic performance.^14^ The high surface area and intrinsic porosity of CTFs make them excellent candidates for energy storage applications. For example, niobium-doped CTFs have demonstrated significant potential in supercapacitors, exhibiting enhanced energy storage performance.^15^ Additionally, CTFs have demonstrated remarkable efficiency in the photocatalytic aerobic oxidation of sulfides, underscoring their potential in organic synthesis. Moreover, the capability to fine-tune their band structures through molecular engineering further enhances their catalytic performance in such reactions.^16^ By capitalizing on their structural versatility and tunable physicochemical properties, researchers continue to explore and expand the potential applications of CTFs across diverse technological and industrial domains.^17^

The synthesis of CTFs has undergone continuous advancement, resulting in significant improvements in synthetic methodologies. The concept of CTFs was first introduced in the late 2000s, when researchers identified their distinctive features, such as aromatic carbon–nitrogen linkages (triazine units) and the absence of weak chemical bonds.^13^ The initial synthesis of CTFs was primarily achieved through ionothermal polymerization, in which molten zinc chloride served as both the solvent and the catalyst. However, this approach typically required temperatures exceeding 300 °C, which often resulted in undesirable carbonization of the polymeric backbone.^18^

In recent years, significant advancements have been made in the synthesis of CTFs, including the development of ionothermal methods operable at lower temperatures. In particular, the use of a ternary eutectic mixture composed NaCl–KCl–ZnCl_2_ with a melting point as low as 200 °C has enabled the synthesis of CTFs under milder conditions. This approach effectively minimizes carbonization and enhances the optical and electronic properties of the resulting materials.^19^ A significant amount of work has focused on enhancing the crystallinity of CTFs, a factor that is crucial in determining their performance across various applications. Accordingly, multiple strategies have been proposed and developed to improve the crystalline quality of these materials.^8^ Ionic liquid catalysts (ILCs) represent a class of catalysts that employ ionic liquids as reaction media to facilitate chemical transformations. Ionic liquids salts that exist in a liquid state at or near ambient temperature exhibit unique characteristics, including negligible volatility, high thermal stability, and adjustable solubility. These characteristics render them highly effective and versatile for a wide range of catalytic applications. The applications of ionic liquids encompass a broad range of chemical processes, including oxidation reactions,^20^ biodiesel production,^21^ CO_2_ hydrogenation,^22^ supported ionic liquid phases,^23^ the Beckmann rearrangement,^24^ and CO_2_ fixation.^25^ Bromine, a halogen element, possesses substantial industrial significance, particularly in pharmaceutical production,^26^ water treatment processes,^27^ and chemical synthesis.^26^ The immobilization of bromine on the surfaces of heterogeneous catalysts is crucial for enhancing catalytic efficiency and stability in a variety of chemical processes. This approach enables the controlled attachment of bromine species to catalyst surfaces, thereby markedly improving the selectivity and reactivity of brominated compounds during reactions. Moreover, anchoring bromine to solid supports substantially reduces the risk of exposure and environmental contamination.

Benzimidazoles are heterocyclic organic compounds consisting of fused benzene and imidazole rings. They exhibit a wide range of applications in medicinal chemistry owing to their diverse biological activities and pharmacological properties.^28–31^ The structural framework of benzimidazoles permits various substitutions on the benzene and imidazole rings, facilitating the synthesis of numerous derivatives with distinct characteristics. These substitutions can have a significant impact on the electronic properties and chemical reactivity of the compounds.^32^ Benzimidazoles have been extensively investigated for their potential as antitubercular agents, with certain derivatives demonstrating promising inhibitory effects against *Mycobacterium tuberculosis* through the targeting of essential proteins such as FtsZ.^28^ Additionally, their antioxidant and antibacterial activities have been documented,^29^ and their efficacy in corrosion protection of mild steel in acidic environments has been examined.^33^ The structural versatility of benzimidazoles has facilitated the development of diverse synthetic methodologies, including green chemistry strategies and the application of nano-based catalysts, aimed at enhancing both the efficiency and selectivity of their synthesis.^30,34^ The utilization of aldehyde precursors in the synthesis of benzimidazoles represents a significant advancement in organic chemistry, facilitating the production of these important heterocyclic compounds. Given the diverse biological activities of benzimidazoles, their synthesis remains a critical area of research. Aldehydes function as key intermediates in various synthetic pathways for benzimidazoles. Notably, the reductive coupling of *ortho*-dinitroarenes with aldehydes, employing molecular hydrogen in the presence of a catalyst, provides a straightforward route to a broad range of functionalized 2-substituted benzimidazoles. This method is particularly advantageous due to its tolerance of various functional groups, underscoring the versatility of aldehyde precursors in efficiently generating complex structures.^35,36^

The incorporation of aldehyde precursors in benzimidazole synthesis not only improves reaction efficiency and yield but also enables the development of novel compounds with significant biological activities. As research progresses, the continued exploration of aldehyde-based strategies is expected to foster further innovations in the synthesis of benzimidazole derivatives, reinforcing their relevance in pharmaceutical applications.

In recent years, a variety of catalysts and methods have been explored for the synthesis of benzimidazoles, each exhibiting specific limitations. For instance, the use of the moisture-sensitive Ph_3_BiCl_2_ necessitates strictly anhydrous conditions, which restricts its practical application; nevertheless, it offers high catalytic activity and excellent selectivity under those controlled conditions.^37^ Similarly, Bi(NO_3_)_3_·5H_2_O presents challenges related to moisture sensitivity, as well as difficulties in recovery and reuse, although it is commercially available, cost-effective, and environmentally benign due to its low toxicity.^38^ The application of MgO@DFNS has also been associated with prolonged reaction times, yet it demonstrates remarkable catalytic performance, under mild and eco-friendly conditions.^39^ These limitations have intensified the pursuit of sustainable and eco-friendly approaches for benzimidazole synthesis. In this study, a straightforward and efficient strategy has been developed to synthesize a wide range of benzimidazoles.

In recent years, nitrogen sources have been used for the synthesis of some organic compounds. In a related context, Li and co-workers recently developed a catalyst- and external hydrogen-free protocol for the synthesis of *N*-(un)substituted lactams, a class of nitrogen-containing heterocycles, directly from oxocarboxylic acids. Their approach relies on the *in situ* release of formic acid from formamides (*e.g.*, H_2_NCHO) or ammonium formate (HCOONH_4_) as the nitrogen source, facilitated by water, which also suppresses undesirable by-product formation. This work underscores the potential of simple nitrogen-rich precursors in enabling sustainable and efficient construction of N-heterocyclic frameworks under mild conditions.^40^ Also, shown in another study demonstrated that ammonium formate (HCOONH_4_) can serve as a dual hydrogen and nitrogen source for the reductive cycloamination of bio-based levulinic acid to 5-methyl-2-pyrrolidone, achieving over 90% yield within 60 min at 180 °C in the absence of any catalyst or additive. Pressurized hot water was found to remarkably enhance the reaction efficiency by promoting the hydrolysis of HCOONH_4_ to liberate ammonia and formic acid for cascade reactions. This catalyst-free protocol was also applicable to the efficient synthesis of various cyclic amides from relevant keto acids.^41^ In addition, in another experiment, developed a benign and eco-friendly approach using low-cost formamide (FAM) as the nitrogen source and formic acid (FA) as the hydrogen source for the synthesis of 5-methyl-2-pyrrolidone from levulinic acid under quasi-catalytic and solvent-free conditions, achieving up to 93% yield within 90 min at 160 °C. Deuterium-labeled experiments and computational studies revealed a transfer hydroamination pathway, where the initial C–N bond formation was the key step contributing to rapid substrate conversion, while the concurrent amidation process facilitated subsequent cyclization to afford the desired lactam. This quasi-catalytic system was also applicable to the synthesis of various *N*-unprotected lactams from keto acids in 76–95% yields under benign conditions.^42^

This study presents the design and synthesis of a tribromide ionic liquid-based covalent triazine framework ([IL-BD-AT]^+^Br_3_^−^) as a heterogeneous and highly efficient catalyst for this application. The introduction of bromine onto the [BD-AT] substrate is pivotal in enhancing the catalytic performance. This modification not only reinforces structural stability but also generates essential active sites for catalysis. Importantly, the bromine species on the surface of [BD-AT] are environmentally benign, rendering the catalyst suitable for sustainable chemical processes. The bromine-doped [BD-AT] framework affords an optimized catalytic environment, facilitating rapid and efficient benzimidazole synthesis.

This work underscores the potential of bromine-functionalized [BD-AT] in heterogeneous catalysis and provides a promising pathway for the development of efficient and sustainable catalytic systems applicable to a broad range of chemical transformations. Our findings underscore the significance of structural design and environmentally conscious considerations in maximizing catalytic efficiency under mild reaction conditions.

**Fig. 1:**
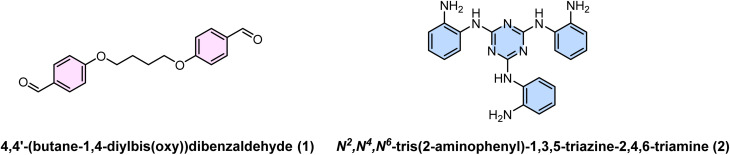
Starting materials used in CTF synthesis.

**Scheme 1:**
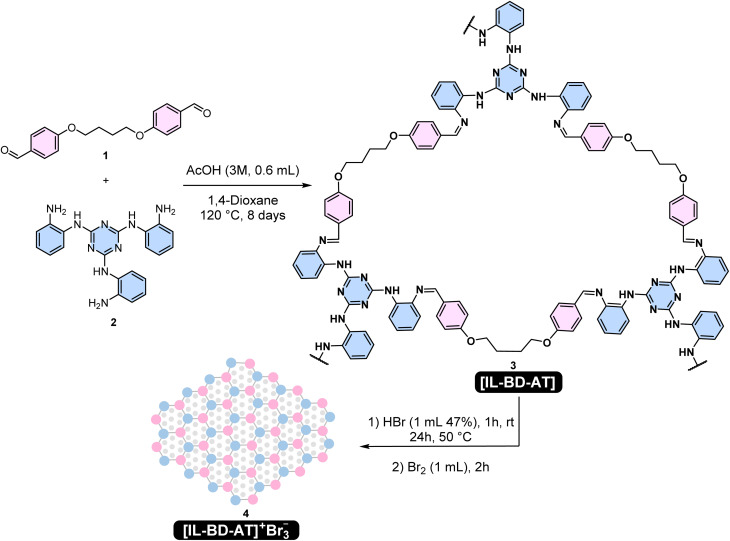
Structure of [IL-BD-AT]^+^Br_3_^−^ (4).

Accordingly, the main goal of the present study is to design and synthesize a novel heterogeneous [IL-BD-AT]^+^Br_3_^−^, and to evaluate its catalytic performance in the green synthesis of benzimidazole derivatives. To achieve this goal, a series of works have been systematically carried out. For example, synthesis of the 4,4′-(butane-1,4-diylbis(oxy))dibenzaldehyde (1), and the *N*^2^,*N*^4^,*N*^6^-tris(2-aminophenyl)-1,3,5-triazine-2,4,6-triamine (2) (Fig. 1), construction of the covalent triazine framework ([BD-AT]) *via* solvothermal condensation (3), immobilization of tribromide species onto the framework to generate the active catalyst [IL-BD-AT]^+^Br_3_^−^ (4) (Scheme 1), comprehensive characterization of the catalyst using FT-IR, EDX, FE-SEM, TEM, BET, TGA, XRD, and XPS techniques; and investigation of the catalytic activity, reusability, and substrate scope of the prepared catalyst in the condensation of various aldehydes with 1,2-phenylenediamine under mild and aqueous conditions, followed by proposal of a plausible reaction mechanism.

## 2. Experimental

### 2.1. Materials

All chemicals were obtained from Merck and Fluka and used as received without further purification. ^1^H and ^13^C NMR spectra were recorded on a 500 MHz Bruker spectrometer in DMSO-d_6_, with chemical shifts reported in ppm. FT-IR spectra were collected using a Shimadzu 435-U-04 spectrophotometer. Energy-dispersive X-ray (EDX) analysis was conducted using a MIRA II instrument (France), and elemental mapping was performed with a MAPPING SAMX system. Field emission scanning electron microscopy (FE-SEM) images were acquired using a MIRA III microscope, while high-resolution transmission electron microscopy (HR-TEM) was carried out on an FEI TECNAI F20. Brunauer–Emmett–Teller (BET) surface area measurements were obtained using a BELSORP Mini II analyzer. X-ray diffraction (XRD) patterns were recorded on a Philips PW1730 diffractometer. Thermogravimetric analysis (TGA) was conducted using a Q600 instrument (USA), and X-ray photoelectron spectroscopy (XPS) measurements were performed on a SpecsLab Prodigy system (Version 4.93.1 r106777).

### 2.2. Catalyst preparation

#### 2.2.1. Preparation of 4,4″-(butane-1,4-diylbis(oxy))dibenzaldehyde (1)

In a round-bottom flask, cyanuric chloride (1 mmol, 0.184 g) was dissolved in acetone (10 mL) and placed in an ice bath to cool. A mixture of water and ice (10 mL) was then added, resulting in a white suspension. Separately, 1,2-phenylenediamine (3.2 mmol, 0.340 g) and sodium hydroxide (3.2 mmol, 0.128 g) were dissolved in a 1 : 1 (v/v) mixture of acetone and water. This solution was added intermittently to the reaction mixture. The resulting mixture was stirred at 0 °C for 2 h, then allowed to reach room temperature and stirred for an additional 2 h, before being refluxed at 60 °C for 18 h. The reaction progress was monitored using thin-layer chromatography (TLC). Upon completion of the reaction, the precipitated product was collected by filtration, washed with cold water, and dried at 100 °C. The structure of the synthesized compound was confirmed through FT-IR, NMR, and mass spectrometric analyses.

#### 2.2.2. Preparation of *N*^2^,*N*^4^,*N*^6^-tris(2-aminophenyl)-1,3,5-triazine-2,4,6-triamine (2)

First, in a 500 mL flask, 1,4-dibromobutane (20 mmol, 2.22 mL), 4-hydroxybenzaldehyde (40 mmol, 4.88 g), and KOH (40 mmol, 2.24 g) were dissolved in acetonitrile (250 mL) and stirred at 80 °C for 12 h. Upon completion of the reaction, the solvent was evaporated, and the residue was extracted with dichloromethane and water. After evaporating the dichloromethane, the product was crystallized from a mixture of water and ethanol. The structure of the obtained compound 2 was confirmed through FT-IR, NMR, and mass spectrometric analyses.^43^

#### 2.2.3. Synthesis of covalent triazine framework ([BD-AT]) (3)

A mixture of 4,4′-(butane-1,4-diylbis(oxy))dibenzaldehyde (1) (3 mmol, 0.89 g) and *N*^2^,*N*^4^,*N*^6^-tris(2-aminophenyl)-1,3,5-triazine-2,4,6-triamine (2) (2 mmol, 0.79 g) was prepared in 1,4-dioxane (40 mL) containing acetic acid (3 M, 0.6 mL) within a Pyrex reaction tube. The resulting suspension was subjected to ultrasonication for 1 h to ensure uniform dispersion of the reactants. Subsequently, the reaction mixture was cooled in a liquid nitrogen bath at 77 K and degassed using three consecutive freeze–pump–thaw cycles to remove dissolved gases. Following degassing, the mixture was transferred to a stainless-steel autoclave equipped with a Teflon liner and heated in an oven at 120 °C for 8 days to facilitate the reaction under solvothermal conditions. Upon completion, a brown precipitate formed, which was isolated by filtration. The solid product was thoroughly washed with *N*,*N*′-dimethylformamide (3 × 10 mL) and tetrahydrofuran (3 × 10 mL) to remove unreacted starting materials and byproducts. Finally, the obtained powder was dried in a vacuum oven at 80 °C for 24 h, yielding the purified product.

#### 2.2.4. Anchoring of bromine on covalent triazine framework [IL-BD-AT]^+^Br_3_^−^ (4)

In a 50 mL round-bottom flask, hydrobromic acid (HBr, 47%, 1 mL) and covalent triazine framework 3 (1.00 g) were stirred at room temperature for 1 h. The reaction mixture was subsequently heated to 50 °C and maintained under continuous stirring for 24 h to afford covalent triazine framework bromide 4. In the subsequent step, bromine (Br_2_, 19.5 mmol) was added to the mixture, as previously described,^44^ and the reaction was allowed to proceed under stirring for 2 h to form the ionic liquid covalent triazine framework tribromide. The resulting catalyst was then washed with a water–ethanol mixture (20 : 20 mL) and dried in an oven at 90 °C for 24 h (Scheme 1).

### 2.3. Catalytic test

Aldehyde 6 (1 mmol, 0.1 mL), 1,2-phenylenediamine 5 (1 mmol, 0.10 g), [IL-BD-AT]^+^Br_3_^−^4 (0.01 g), and H_2_O (5 mL) were added to a round-bottom flask. The reaction mixture was stirred at 80 °C for 5 min. The catalyst was separated from the mixture by centrifugation, and the resulting product was purified by silica gel plates using *n*-hexane and ethyl acetate (40 : 10). The obtained product was extensively characterized by FT-IR, ^1^H NMR, and ^13^C NMR analyses.

## 3. Results and discussion

### 3.1. Characterization of ionic covalent triazine frameworks

**Fig. 2:**
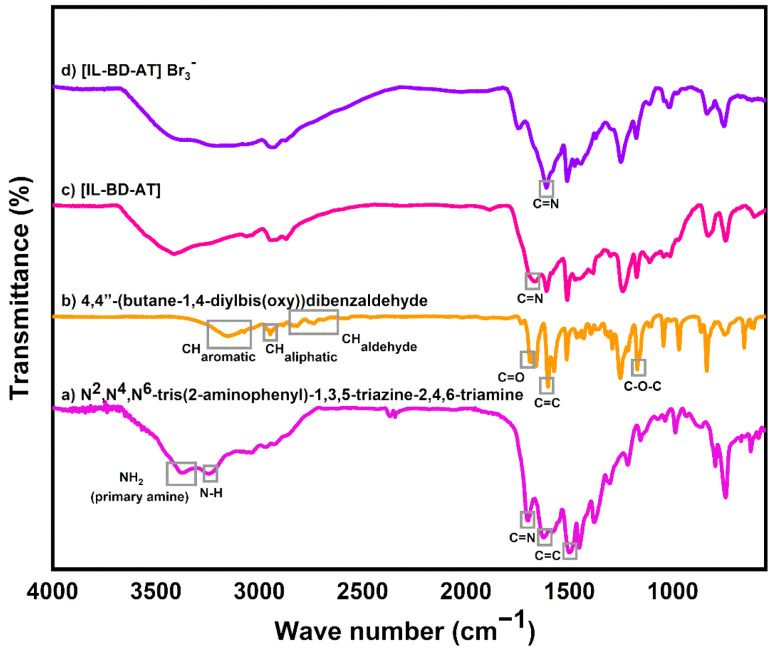
FT-IR spectra: (a) *N*^2^,*N*^4^,*N*^6^-tris(2-aminophenyl)-1,3,5-triazine-2,4,6-triamine 2, (b) 4,4″-(butane-1,4-diylbis(oxy))dibenzaldehyde 1, (c) [BD-AT] 3, (d) ([IL-BD-AT]^+^Br_3_^−^) 4.

The starting materials, [BD-AT] 3 and [IL-BD-AT]^+^Br_3_^−^ were analyzed using Fourier transform infrared (FT-IR) spectroscopy (Fig. 2). In the FT-IR spectrum of *N*^2^,*N*^4^,*N*^6^-tris(2-aminophenyl)-1,3,5-triazine-2,4,6-triamine 2 (Fig. 2a), the peaks at 3375 cm^−1^ and 3245 cm^−1^ correspond to NH_2_ and NH groups, respectively. The peak at 3050 cm^−1^ is attributed to the aromatic C–H stretching vibration. C=N stretching vibrations appear at 1699 cm^−1^. The peaks at 1623 cm^−1^ and 1453 cm^−1^ are assigned to aromatic C=C stretching vibrations. In the FT-IR spectrum of 4,4″-(butane-1,4-diylbis(oxy))dibenzaldehyde 1 (Fig. 2b), the stretching vibrations at 3154 cm^−1^ and 2946 cm^−1^ correspond to aromatic C–H and aliphatic C–H, respectively, while the stretching vibrations at 2816 cm^−1^ and 2733 cm^−1^ are associated with the aldehyde C–H. The aromatic C=O and C=C stretching vibrations appear at 1691 cm^−1^ and 1601 cm^−1^, respectively. The peak at 1171 cm^−1^ corresponds to the ether functionality of the compound. In the FT-IR spectrum of [IL-BD-AT] (Fig. 2c), although the absorption band of the newly formed C=N bond resulting from the condensation of the aldehyde and amine cannot be clearly observed, the reduction of the NH_2_ peak indicates a significant level of polymerization. Finally, Br_3_ exhibits a signal in the range of 180–200 cm^−1^, which cannot be detected in the FT-IR spectrum (Fig. 2d).^45^ The absence of a clear Br_3_ signal in the FT-IR spectrum aligns with prior reports where tribromide vibrations lie beyond the typical IR spectral range.

**Fig. 3:**
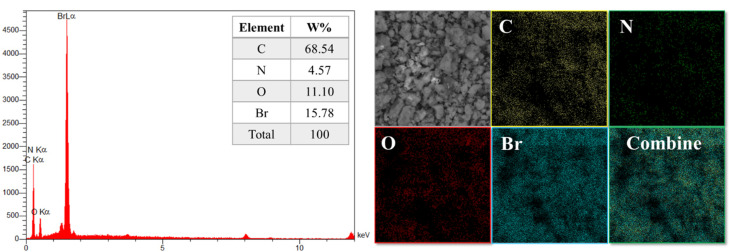
EDX and elemental mapping images of ([IL-BD-AT]^+^Br_3_^−^) 4.

Energy-dispersive X-ray (EDX) spectroscopy confirms the presence of key constituent elements within the material, including carbon (35.4 wt%), nitrogen (2.75 wt%), oxygen (7.64 wt%), and bromine (54.21 wt%) (Fig. 3). The notably high bromine content indicates successful and efficient tribromide loading on the [BD-AT] 3 surface, exceeding typical bromine incorporation levels reported in similar systems. This elemental distribution validates the intended functionalization strategy and demonstrates that bromine anchoring does not adversely affect the framework's overall elemental composition. The substantial bromine presence is particularly significant, as it represents the active catalytic site in the system and is therefore essential for the material's catalytic performance. Elemental mapping images demonstrate a uniform distribution of C, N, O, and Br across the [IL-BD-AT]^+^Br_3_^−^ framework (Fig. 3). This homogeneous spatial dispersion suggests that the bromine species are effectively integrated within the CTF matrix, thereby preventing agglomeration or phase separation. Comparable uniformity in elemental mapping has been reported to contribute to improved catalyst stability and active site accessibility in similar porous materials. Accordingly, the mapping results not only validate the successful immobilization of bromine but also corroborate the potential for high catalytic performance due to readily accessible active sites.

**Fig. 4:**
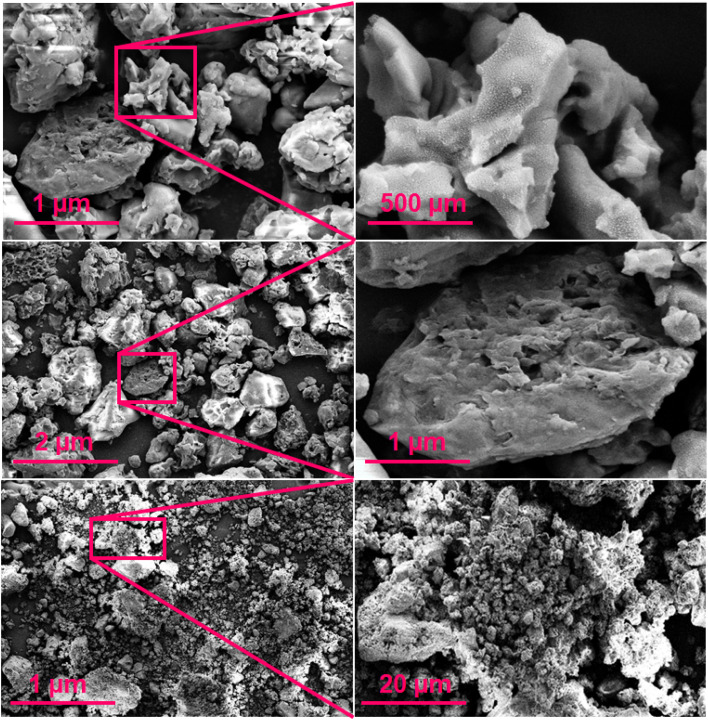
FE-SEM images of ([IL-BD-AT]^+^Br_3_^−^) 4.

Field Emission Scanning Electron Microscopy (FE-SEM) analysis provides essential insights into the surface morphology and structural characteristics of the [IL-BD-AT]^+^Br_3_^−^4 catalyst (Fig. 4). The FE-SEM images reveal a distinctive coral-like structure, indicating a highly porous and irregular surface topology that may facilitate enhanced catalytic activity.

**Fig. 5:**
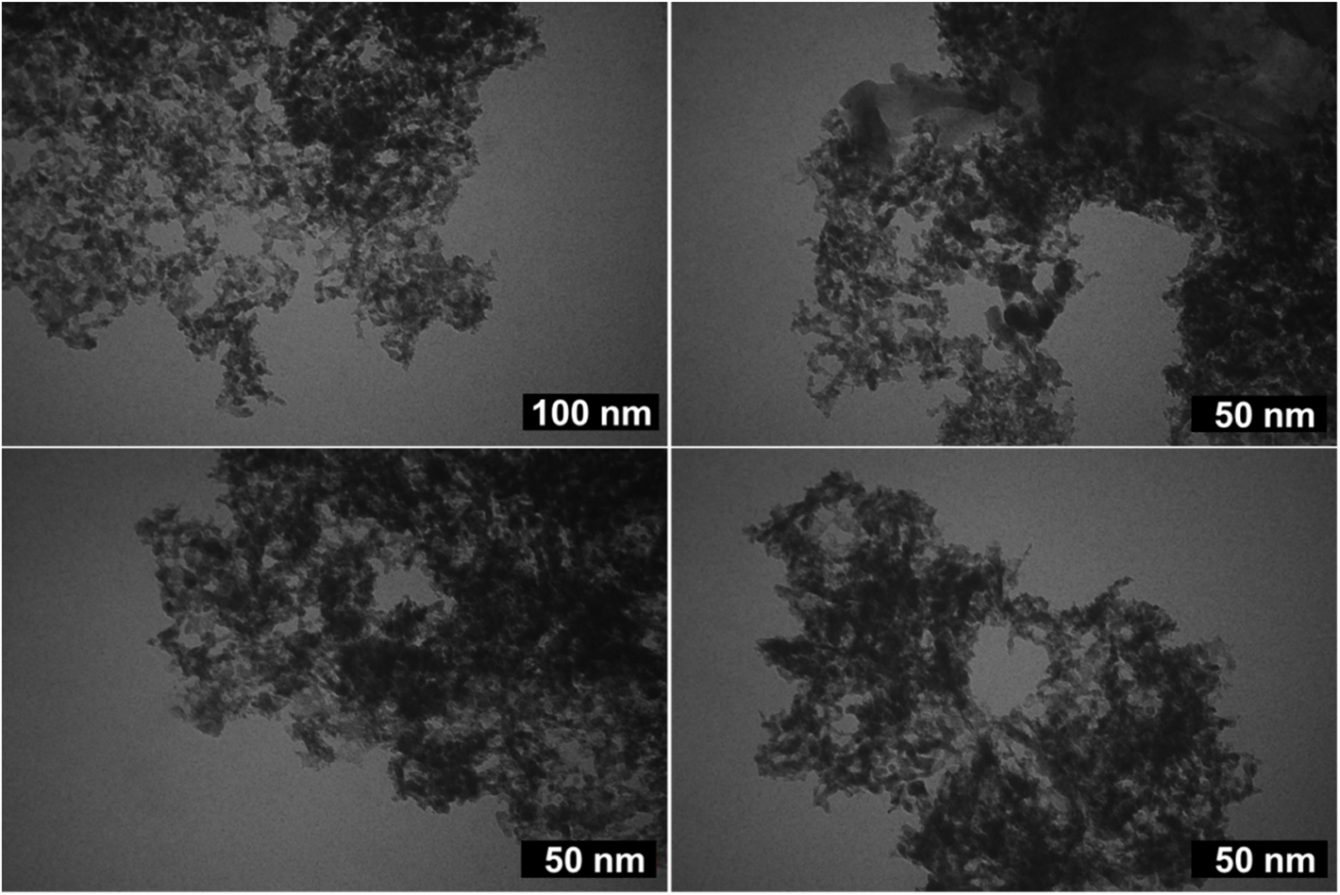
TEM images of [IL-BD-AT]^+^Br_3_^−^ (4).

Transmission electron microscopy (TEM) analysis provides valuable insights into the nanostructure and morphological features of the [IL-BD-AT]^+^Br_3_^−^4 catalyst (Fig. 5). This advanced imaging technique enables direct visualization of the material's internal architecture, offering detailed information on the arrangement of structural units and the distribution of bromine atoms on the [BD-AT] 3 surface. In some areas of the images, bromine atoms may appear as dark spots or localized areas of enhanced electron density on the surface, providing visual evidence of successful bromine functionalization. The observed distribution of bromine atoms further indicates that these functional groups are well-dispersed and accessible on the material's surface. This accessibility is critical for promoting effective interactions with reactant molecules, thereby enhancing the catalyst's potential performance in chemical transformations or adsorption processes.

**Fig. 6:**
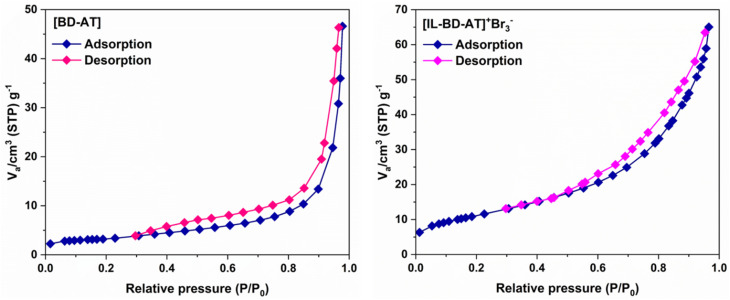
Adsorption and desorption isotherms of [BD-AT] 3, and [IL-BD-AT]^+^Br_3_^−^4, adsorptive N_2_ and adsorption temperature at 77 K.

The porosity and surface area of [BD-AT] 3, and [IL-BD-AT]^+^Br_3_^−^4 were characterized using nitrogen adsorption–desorption analysis conducted at 77 K. The corresponding adsorption and desorption isotherms are presented in Fig. 6, with N_2_ employed as the adsorptive gas under these conditions. Based on the Brunauer–Emmett–Teller (BET) method, the specific surface area of [BD-AT] 3, and [IL-BD-AT]^+^Br_3_^−^ were determined to be 645, and 673 m^2^ g^−1^. The total pore volume (*V*_p_), calculated at a relative pressure of *P*/*P*_0_ = 0.99, were founds to be 0.63 cm^3^ g^−1^, and 0.45 for [BD-AT] 3, and [IL-BD-AT]^+^Br_3_^−^4, respectively. Furthermore, the mean pore diameter, estimated using the BET equation (*r* = 2*V*_p_/*S*), was calculated to be 7.6 nm for [BD-AT] 3, and 5.3 nm for [IL-BD-AT]^+^Br_3_^−^4. Complementary Barrett–Joyner–Halenda (BJH) analysis revealed an average pore width of 2.71 nm, and 2.51 nm for [BD-AT] 3, and [IL-BD-AT]^+^Br_3_^−^4, respectively, this confirms the presence of mesoporous structures. These findings demonstrate that [IL-BD-AT]^+^Br_3_^−^4 possesses a high surface area and well-developed porosity, which are advantageous properties for applications in adsorption and catalysis.

**Fig. 7:**
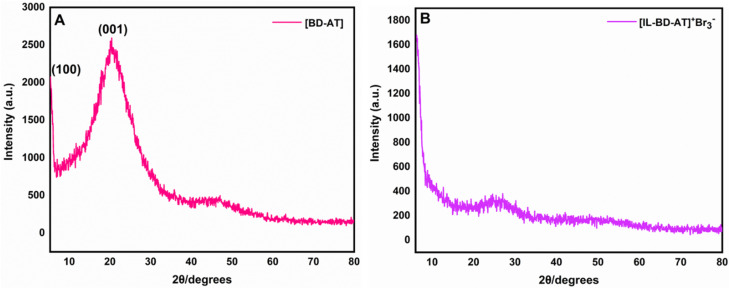
(A) XRD pattern of [BD-AT] 3, and (B) XRD pattern of [IL-BD-AT]^+^Br_3_^−^ (4).

The X-ray diffraction (XRD) analysis indicated the presence of characteristic diffraction peaks at 2*θ* values of 5.39° and 20.4°, corresponding to the (100) and (001) planes, respectively, as shown in Fig. 7A. These peaks suggest the presence of a potentially isoelectronic layered structure.^46,47^ The broad reflection observed at 20.4° indicates a degree of long-range order in the [BD-AT] 3 framework, which may be attributed to its sheet-like morphology. Further structural insights were obtained following surface bromination of [BD-AT] 3, as illustrated in Fig. 7B. Notably, a pronounced decrease in the intensity of the characteristic peaks at 2*θ* = 5.39° and 20.4° was observed. These peaks are typically associated with the ordered pore structure and long-range periodicity of the material. The attenuation of these reflections suggests that bromination induces significant structural alterations within the framework of [BD-AT] 3. Such modifications may perturb both the long-range order and the local atomic arrangement, potentially impacting the material's physicochemical properties and performance in catalytic or adsorption applications.

**Fig. 8:**
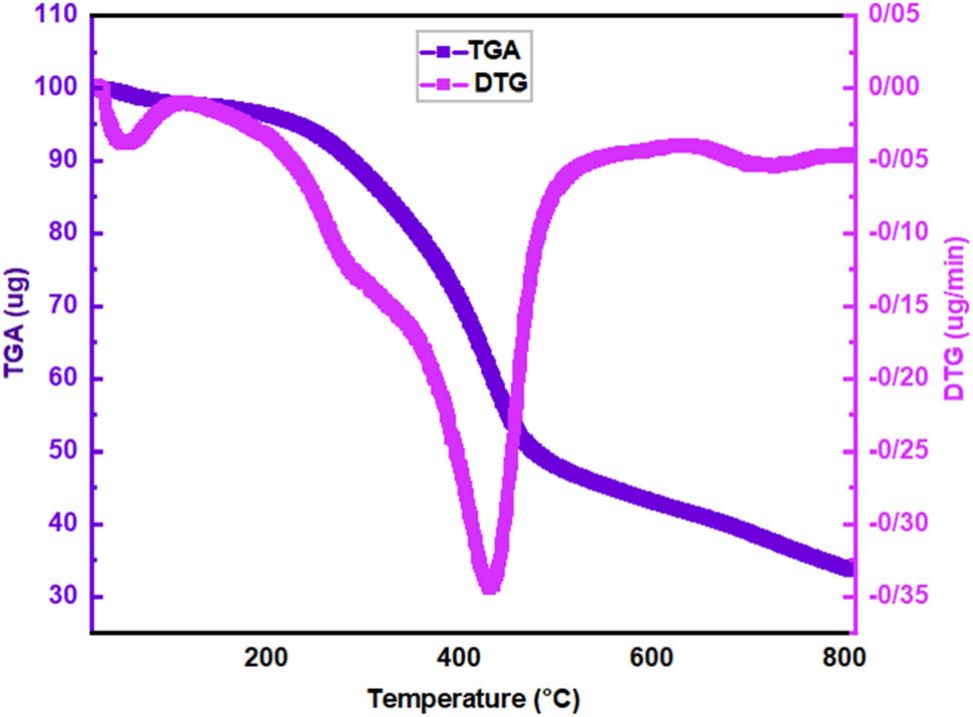
TG-DTG graph of [IL-BD-AT]^+^Br_3_^−^ (4).

Thermogravimetric analysis (TGA) was conducted to assess the thermal stability of the compound. The measurement was performed under a nitrogen atmosphere, with the temperature increased from 25 °C to 800 °C at a heating rate of 10 °C min^−1^ (Fig. 8). According to the derivative thermogravimetric (DTG) analysis, the thermal decomposition process can be divided into four distinct stages of mass loss. The first weight loss, occurring between 25 °C and 60 °C, is attributed to the removal of physically adsorbed water and residual solvents. The second stage of weight loss is observed at approximately 288 °C, corresponding to the initial degradation of chemical bonds within the material. This indicates the onset of decomposition of specific structural components of the CTF. The most significant mass reduction takes place around 430 °C, representing the significant structural degradation of the catalyst. The final decomposition phase is observed at approximately 715 °C, where the remaining framework structure breaks down completely. Overall, the TGA results demonstrate that the catalyst exhibits considerable thermal stability. This is evidenced by its ability to retain structural integrity up to high temperatures, suggesting its suitability for catalytic applications under thermally demanding conditions.

**Fig. 9:**
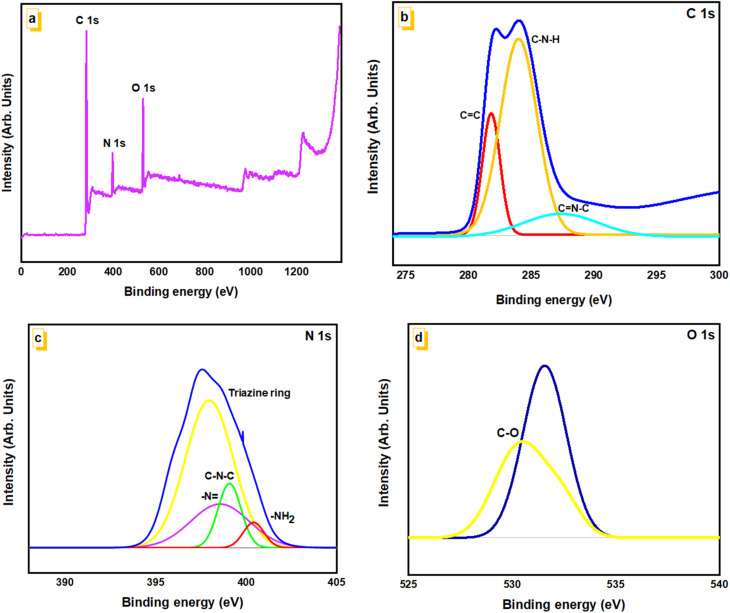
XPS analysis of [IL-BD-AT] (3): (a) survey XPS spectra, high-resolution XPS spectra of (b) C 1s, (c) N 1s, (d) O 1s.

Element-specific chemical analysis of [IL-BD-AT] was performed using X-ray photoelectron spectroscopy (XPS), and the corresponding results are presented in Fig. 9. The survey scan of [IL-BD-AT] (Fig. 9a) reveals three prominent binding energy peaks at 283.0, 397.0, and 530.0 eV, corresponding to C 1s, N 1s, and O 1s, respectively.^48^ The high-resolution C 1s spectrum (Fig. 9b) displays four main contributions, with peaks at 281.9, 284.66, and 286.9 eV, which can be attributed to carbon atoms in benzene (C=C), C–N–H, and C=N–C environments, respectively.^49^ The high-resolution N 1s spectrum (Fig. 9c) can be deconvoluted into four peaks at 398.0, 399.54, 400.1, and 401.6 eV, corresponding to nitrogen atoms in the triazine ring, –N = , C–N–C, and dangling –NH_2_ groups, respectively.^48^ The high-resolution O 1s spectrum (Fig. 9d) exhibits a single peak at 531.8 eV, which is attributed to C–O bonds.^50^ These XPS results confirm the successful incorporation of the expected chemical functionalities within the [BD-AT] framework, providing detailed information on the bonding environment of carbon, nitrogen, and oxygen atoms in the material.

### 3.2. Catalytic properties of [IL-BD-AT]^+^Br_3_^−^

This section evaluates the efficacy of [IL-BD-AT]^+^Br_3_^−^4 as a catalyst for benzimidazole production *via* the condensation of 1,2-phenylenediamine 5 and benzaldehyde 6a as model substrates. A comprehensive analysis of various reaction parameters, including solvent choice, catalyst loading, and temperature, was conducted to optimize the yield of the target benzimidazole 7a (Table 1, entries 1–9). To maximize catalytic performance, the model reaction underwent systematic optimization. The study revealed that aqueous conditions, coupled with a minimal catalyst loading of 0.01 g at 80 °C under an air atmosphere. The optimized reaction conditions yielded the most favorable results (Table 1, entry 4). This finding underscores the synergistic effect of water as a green solvent and low catalyst loading in enhancing the efficiency of benzimidazole 7a synthesis using the [IL-BD-AT]^+^Br_3_^−^4 catalyst. The superior reactivity observed in water relative to ethanol and acetonitrile can be rationalized based on the Acceptor Numbers (AN) of the solvents, as proposed by Gutmann. According to the scale established by Mayer, Gerger, and Gutmann,^51^ water possesses a high AN value of 54.8, which is indicative of its strong electrophilic character. This property enables water to effectively activate the aldehyde carbonyl toward nucleophilic attack. In contrast, ethanol (AN ≈ 37) exhibits moderate electrophilic assistance, whereas acetonitrile, with a low AN of 18.9, provides minimal activation. In the present reaction system, water molecules are still capable of coordinating to the aldehyde oxygen, thereby enhancing the electrophilicity of the carbonyl carbon and facilitating the subsequent condensation with the diamine.

**Table 1:** Systematic optimization of the reaction conditions employed in benzimidazole synthesis^a^

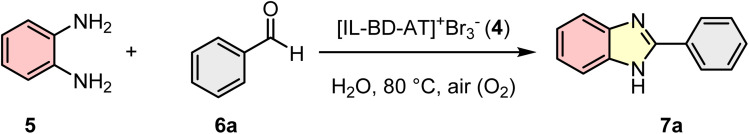					
Entry	Catalyst 4 (g)	Solvent	Time (min)	*T* (°C)	Yield^b^ (%)
1	—	—	30	80	Trace
2	—	H_2_O	30	80	Trace
3	0.006	H_2_O	10	80	50
**4**	**0.01**	**H**_**2**_ **O**	**5**	**80**	**95**
5	0.15	H_2_O	10	80	95
6	0.01	H_2_O	10	rt	53
7	0.01	—	30	80	58
8	0.01	EtOH	10	80	83
9	0.01	CH_3_CN	30	75	47

a Reaction conditions: aldehyde 6a (1 mmol), 1,2-phenylenediamine 5 (1 mmol), catalyst (0.01 g), H_2_O (5 mL), air (O_2_), 80 °C.

b Isolated yields.

The optimized reaction conditions were applied to a diverse array of aldehydes 7a–k in combination with 1,2-phenylenediamine 5 and the [IL-BD-AT]^+^Br_3_^−^4 catalyst. The results indicated that aldehydes bearing both electron-withdrawing and electron-donating substituents exhibited high reactivity, affording improved yields of the target compounds (Table 2). These findings demonstrate the versatility of the [IL-BD-AT]^+^Br_3_^−^4 catalytic system in promoting the efficient synthesis of structurally diverse benzimidazole derivatives.

**Table 2:** Synthesis of various symmetric benzimidazoles^a^

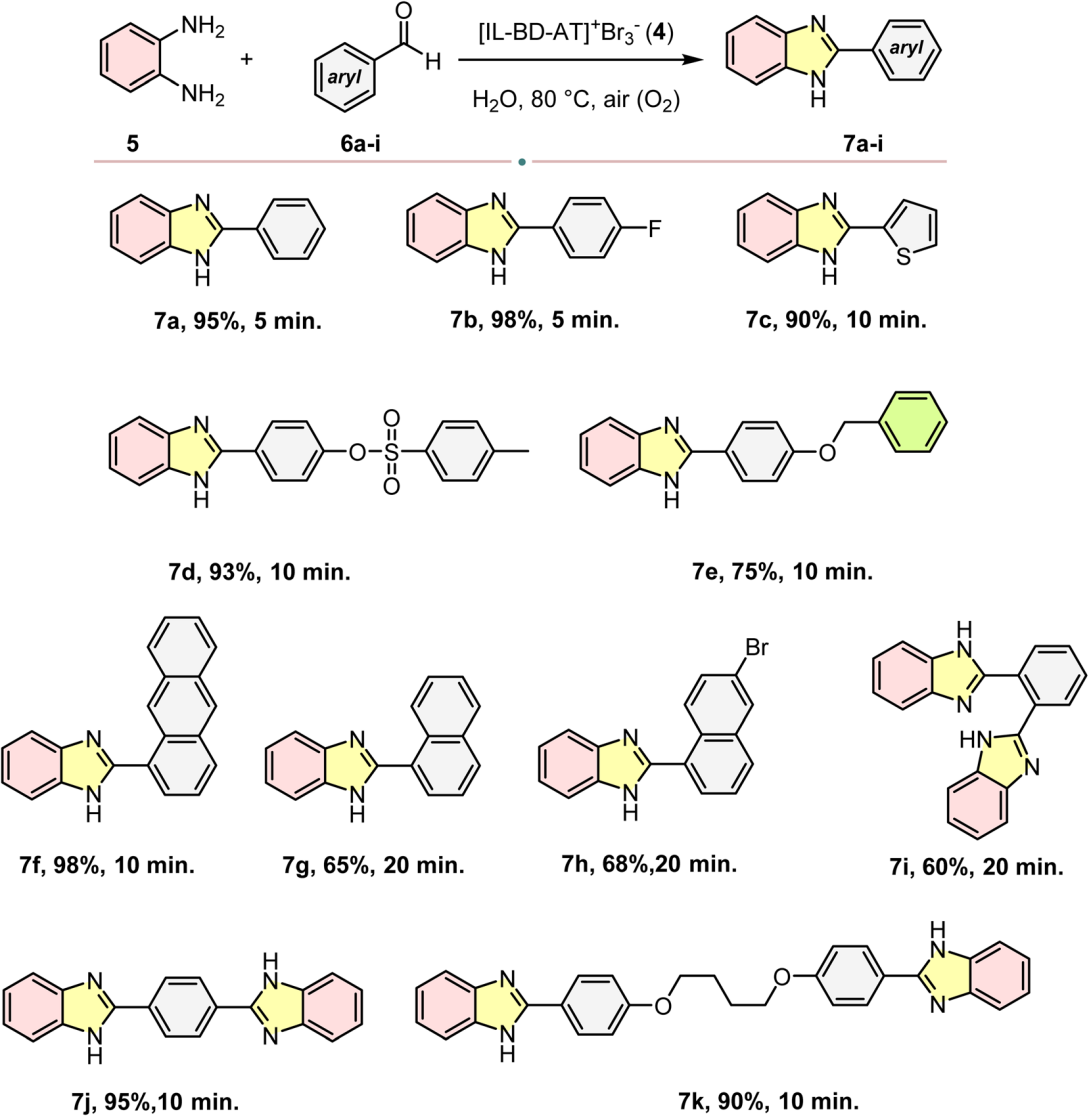

a Reaction conditions: benzaldehyde (1 mmol), 1,2-phenylenediamine (1 mmol), catalyst 4 (0.01 g), H_2_O (5 mL).

### 3.3. Efficiency of ionic covalent triazine frameworks

By immobilizing bromine on the [BD-AT] 3 surface, this method effectively mitigates the hazards associated with free bromine. It significantly reduces the risks of toxicity, corrosiveness, and environmental damage typically linked to bromine handling. The use of water as the reaction solvent offers an environmentally benign alternative to conventional organic solvents, being non-toxic and contributing to the overall sustainability of the process. Performing the reaction under an air atmosphere further simplifies the procedure. This strategy not only enhances accessibility but also aligns with green chemistry principles by minimizing resource consumption. Experimental results demonstrate a marked improvement in reaction efficiency when using [IL-BD-AT]^+^Br_3_^−^4 compared to the unmodified [BD-AT] 3. Considering that the model reaction is conducted at 80 °C, the application of molecular bromine (Br_2_) under these conditions introduces considerable safety risks due to its high volatility and reactivity. Consequently, the execution of this reaction under conventional laboratory conditions is not feasible. *In lieu* of the direct use of Br_2_, the model reaction was performed employing [BD-AT] 3 (Table 3). This enhancement is reflected in higher yields, faster reaction rates, or both, highlighting the catalytic efficacy of [IL-BD-AT]^+^Br_3_^−^4. The immobilized bromine on the [BD-AT] 3 surface plays a key role in promoting benzimidazole formation, likely acting as a Lewis acid to activate the aldehyde and facilitate nucleophilic attack by the amine. Additionally, bromine may assist in the oxidation step, accelerating the formation of the aromatic benzimidazole ring. The combination of [BD-AT] 3's high surface area and porosity with the catalytic activity of surface-bound bromine generates a synergistic effect, enhancing mass transfer, increasing the local concentration of reactive species, and potentially lowering activation energies for critical reaction steps. This approach not only improves benzimidazole synthesis but also provides a general strategy for heterogeneous catalysis.

**Table 3:** Comparison of catalyst completion steps for the synthesis of 2-phenyl-1*H*-benzo[*d*]imidazole

Entry	Catalyst	Time (min)	Yield (%)
1	[BD-AT] 3	20	53
2	[IL-BD-AT]^+^Br_3_^−^4	5	95

The results presented in Table 4 demonstrate that the ionic liquid covalent triazine framework catalyst [IL-BD-AT]^+^Br_3_^−^4 exhibits superior catalytic performance for the synthesis of 2-phenyl-1*H*-benzimidazole compared to a range of reported catalysts in the literature. This catalyst achieves a remarkable yield of 98% within a brief reaction time of 5 to 20 minutes at 80 °C under aqueous conditions, which is significantly shorter and milder than many conventional catalysts that often require prolonged heating times and organic solvents. The high catalytic efficiency can be attributed to the unique structural features of the covalent triazine framework, which provides a high surface area (657.5 m^2^ g^−1^) and mesoporosity, facilitating efficient substrate access to the catalytically active sites. Furthermore, the immobilization of tribromide anions within the framework serves as active Lewis acid sites that enhance the activation of aldehyde substrates, thereby accelerating the condensation reaction to form benzimidazoles. In comparison to catalysts such as A-FGO, Zn(OTf)_2_, and CuO–SiO_2_, the [IL-BD-AT]^+^Br_3_^−^4 catalyst offers both environmental and practical advantages. Its use of water as a solvent aligns well with green chemistry principles, reducing environmental impact by avoiding hazardous organic solvents. Additionally, the heterogeneity of this catalyst allows for facile separation and reuse, maintaining high catalytic activity over multiple cycles as demonstrated in the study. This work contributes significant advancements to the field by demonstrating that the strategic design of covalent triazine frameworks functionalized with ionic liquid tribromide species can overcome limitations observed in previous catalytic systems. The rapid reaction times, high yields, operational simplicity, and green solvent conditions mark important improvements that may facilitate broader adoption of such materials in synthetic organic chemistry. The findings presented reinforce the potential of carefully engineered porous frameworks as multifunctional heterogeneous catalysts and open new avenues for sustainable benzimidazole production under mild conditions.

**Table 4:** Comparison of various catalysts in synthesizing 2-phenyl-1*H*-benzo[*d*]imidazole

Entry	Catalyst	Solvent	Temp. (°C)	Time (min)	Yields (%)	Ref.
1	A-FGO (0.1 g)	THF	Reflux	120	86	52
2	Zn (OTf) (10 mol%)	EtOH	Reflux	8 h	94	53
3	CuO–SiO_2_ (10 mol%)	CH_3_OH	rt	4–8 h	73–96	54
**4**	**[IL-BD-AT]** ^**+**^ **Br**_**3**_ **^−^4 (0.01)**	**H**_**2**_ **O**	**80**	**5**	**95**	**Our work**

#### 3.3.1. Recycling and reusing the catalyst

**Fig. 10:**
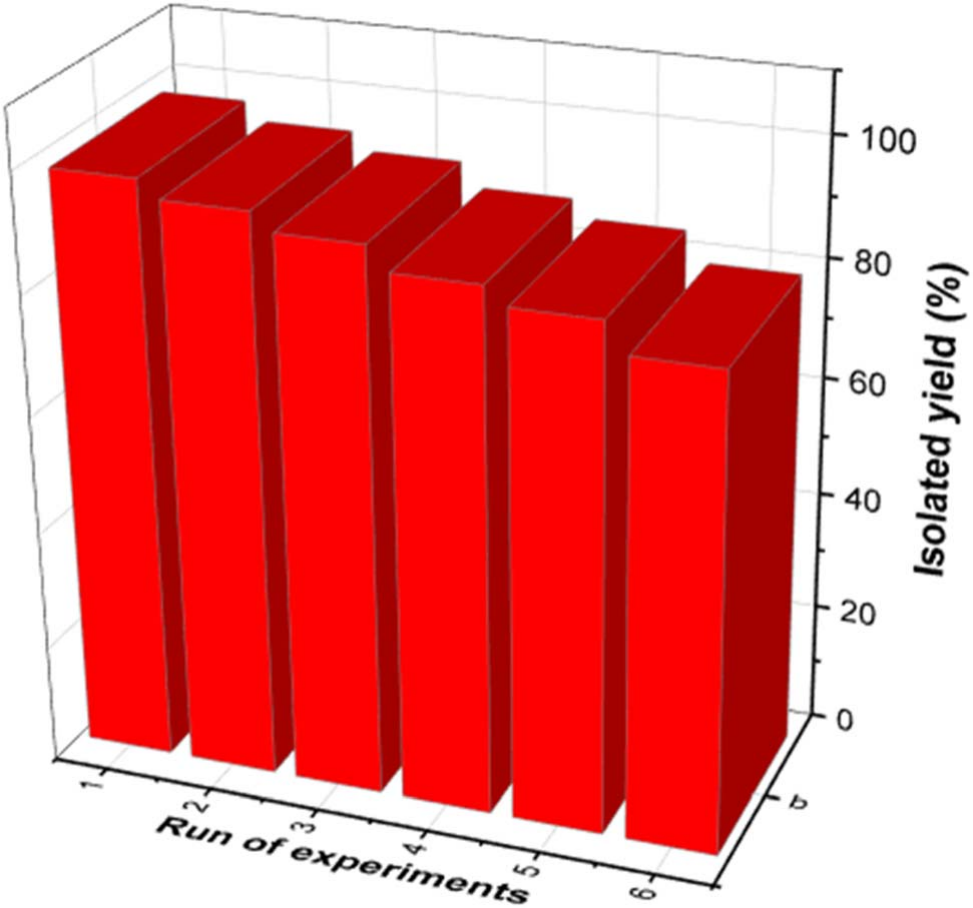
Reusability of the catalyst 4.

To assess the reusability of the [IL-BD-AT]^+^Br_3_^−^4 catalyst, a series of consecutive reactions was conducted using a model system composed of benzaldehyde and 1,2-phenylenediamine (hot filtration test). In each reaction cycle, 0.01 g of catalyst was employed. Following each reaction, the catalyst was recovered by centrifugation, subsequently purified through washing with hot ethanol, and dried for reuse in subsequent cycles. Six consecutive reaction cycles were performed, and the catalyst's performance was evaluated after each iteration. The results indicated that [IL-BD-AT]^+^Br_3_^−^4 retained its catalytic activity over five cycles, exhibiting only a marginal decrease in efficiency, as shown in Fig. 10.

**Fig. 11:**
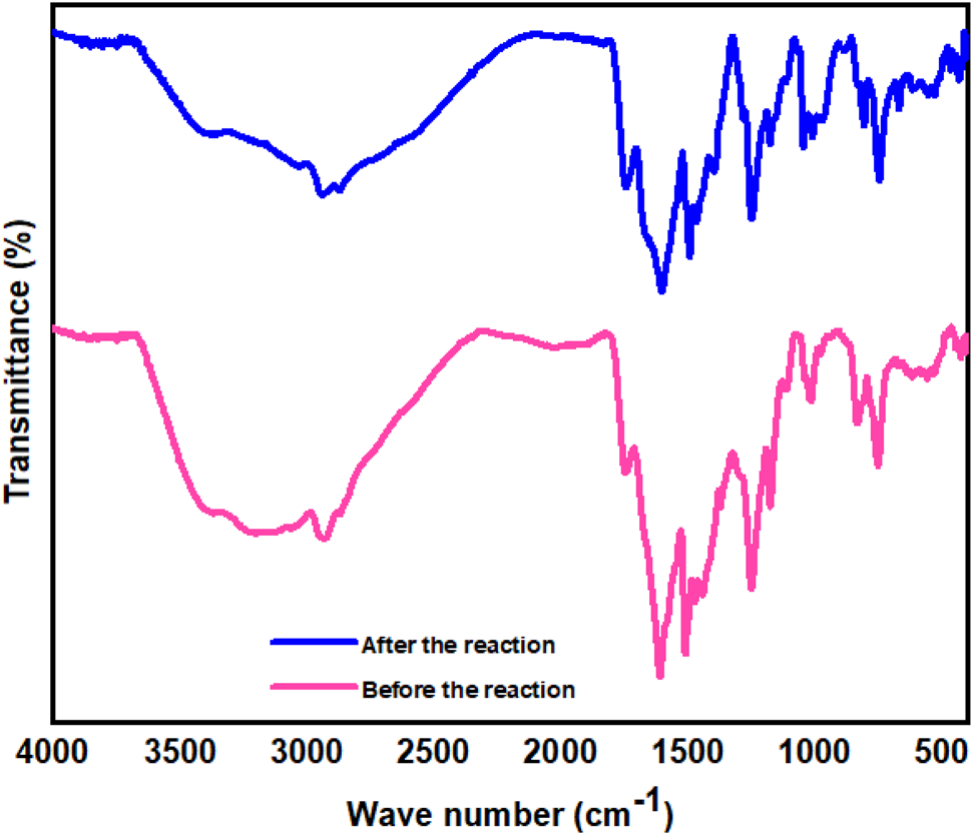
FT-IR spectra of [IL-BD-AT]^+^Br_3_^−^4 before and after the reaction.

**Fig. 12:**
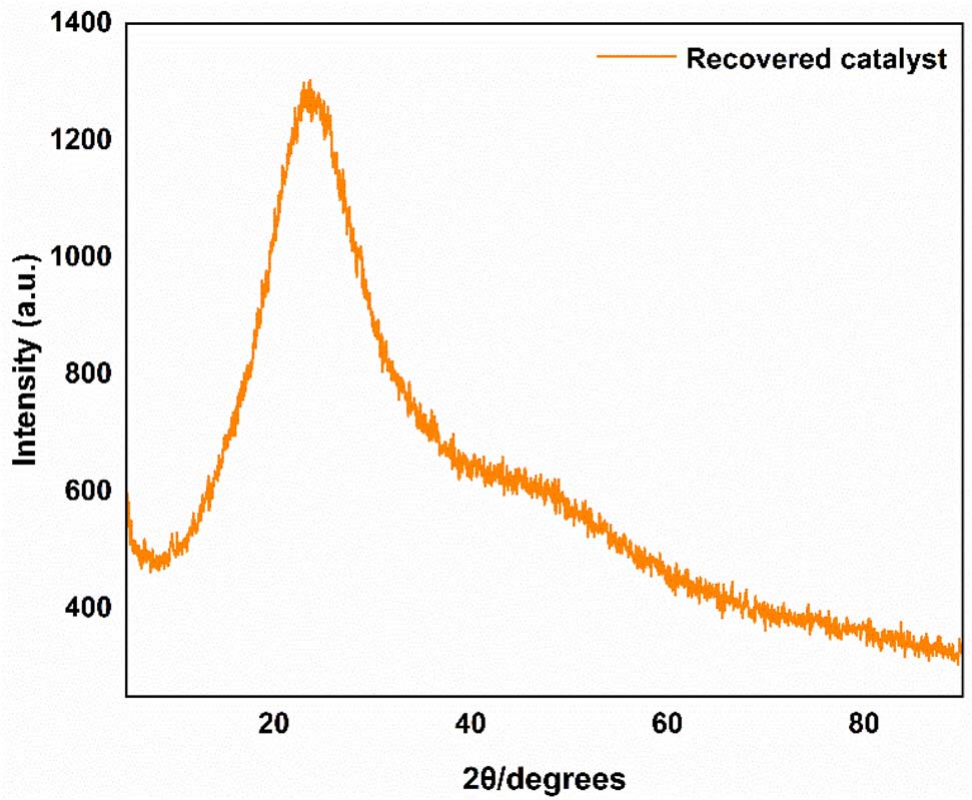
XRD analysis of recovered [IL-BD-AT]^+^Br_3_^−^.

FT-IR spectroscopy analysis of the recovered catalyst revealed slight changes in some absorption bands compared to the fresh catalyst (Fig. 11), indicating minor alterations in surface functional groups during the reaction. To further assess the structural stability, XRD analysis was performed on the recovered catalyst (Fig. 12). The XRD patterns confirm that the structure of catalyst remained essentially unchanged, demonstrating that the catalyst retains its overall integrity despite minor surface modifications. These findings collectively support the catalyst's acceptable stability and reusability.

#### 3.3.2. The proposed reaction mechanism

**Scheme 2:**
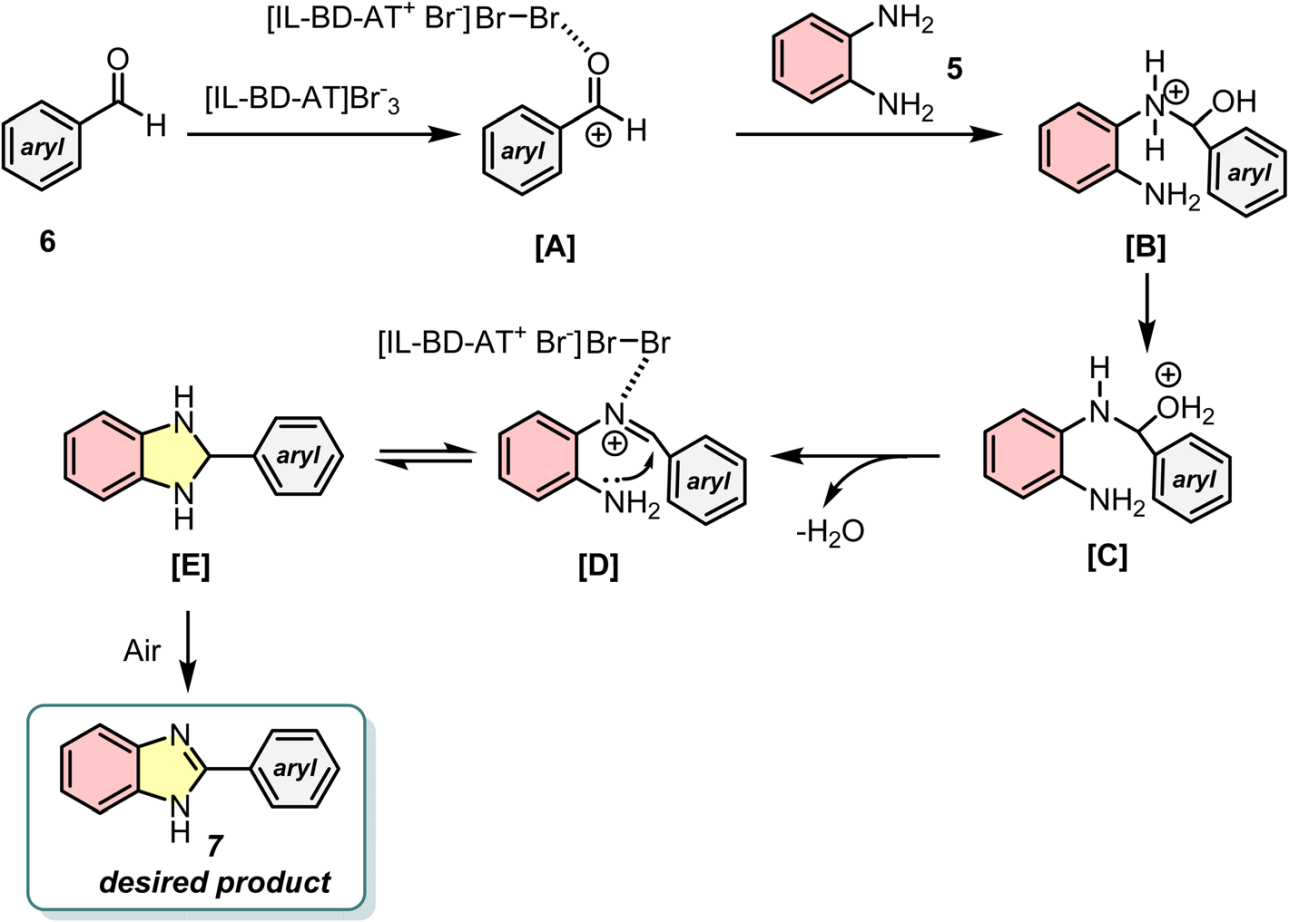
Proposed mechanism for the synthesis of benzimidazoles.

The proposed mechanism for the synthesis of benzimidazoles is depicted in Scheme 2. Initially, the catalyst interacts with the aldehyde, generating a more electrophilic intermediate (A), thereby enhancing the reactivity of the carbonyl group.^55^ The primary amine of 1,2-phenylenediamine subsequently attacks the activated aldehyde in a nucleophilic addition, leading to the formation of intermediate B, a hemiaminal structure. Following this, hydrogen migration occurs, yielding intermediate C. Intermediate C then undergoes dehydration to form an imine bond, resulting in intermediate D. The second amine group of the molecule attacks the imine carbon, triggering intramolecular cyclization and forming intermediate E, a dihydrobenzimidazole structure. The final step involves the oxidation of intermediate E, which can be facilitated by molecular oxygen (air) as a green oxidant. This oxidative process induces dehydrogenation, resulting in the formation of the aromatic benzimidazole ring and completing the synthesis of the desired 2-substituted benzimidazole (7).

## 4. Conclusions

In conclusion, this study represents a significant advancement in the synthesis of benzimidazoles through the innovative application of the tribromide ionic liquid covalent triazine framework [IL-BD-AT]^+^Br_3_^−^4 as a heterogeneous catalyst. The successful incorporation of bromine into the CTF not only enhances its catalytic efficiency but also addresses environmental and safety concerns typically associated with free bromine. The high nitrogen content and structural integrity of [IL-BD-AT]^+^Br_3_^−^4 provide essential active sites for catalysis, enabling the efficient and rapid formation of benzimidazole derivatives under mild reaction conditions. Experimental results demonstrate the superior performance of [IL-BD-AT]^+^Br_3_^−^4 relative to other catalysts reported in the literature, achieving high yields and reaction rates while maintaining stability and recyclability. These findings underscore the potential of covalent triazine frameworks in the development of sustainable and eco-friendly catalytic systems for organic synthesis. Overall, this work contributes to the field of benzimidazole synthesis and opens new avenues for the application of CTFs in diverse chemical transformations. The results highlight the importance of structural engineering and the strategic utilization of environmentally benign materials in catalyst design, thereby paving the way for future research in sustainable chemistry and catalysis.

## Author contributions

Azin Kharazmi: involved in conceptualization, methodology, resources, writing—original draft, reviewing and editing, formal analysis. Ramin Ghorbani-Vaghei: took part in conceptualization, visualization, investigation, supervision, reviewing and editing. Ardeshir Khazaei: supervision. Idris Karakaya: took part in data curation, formal analysis, reviewing and editing. Rahman Karimi-Nami: reviewing and editing, involved in data curation and formal analysis.

## Conflicts of interest

The authors declare that they have no known competing financial interests or personal relationships that could have appeared to influence the work reported in this paper.

## Supplementary Material

RA-016-D6RA04048A-s001

## Data Availability

The supporting information (SI) contains data and characterization (^1^H NMR, ^13^C NMR, and Mass) of synthetic organic compounds. Supplementary information is available. See DOI: https://doi.org/10.1039/d6ra04048a.

## References

[cit1] Anastas P., Eghbali N. (2010). green chemistry: principles and practice. Chem. Soc. Rev..

[cit2] Ivanković A., Dronjić A., Bevanda A. M., Talić S. (2017). review of 12 principles of green chemistry in practice. Int. J. Sustain. Energy.

[cit3] Ch S. L., Sameera M., Jayasree M., Kamala G., Degala R. P. (2025). a review of industrial applications of green chemistry. J. Pharma Res..

[cit4] Ogodo U. P., Abosede O. O. (2025). the role of chemistry in achieving sustainable development goals: green chemistry perspective. Int. Res. J. Pure Appl. Chem..

[cit5] Mane S. R., Bais S. K., Nigadi S. R. (2023). Review on green chemistry and catalysis. Energy.

[cit6] Eskandarinezhad S., Khosravi R., Amarzadeh M., Mondal P., Magalhães Filho F. J. C. (2021). application of different nanocatalysts in industrial effluent treatment: A review. J. Compos. Compd..

[cit7] Ran N., Zhao L., Chen Z., Tao J. (2008). recent applications of biocatalysis in developing green chemistry for chemical synthesis at the industrial scale. Green Chem..

[cit8] Sun R., Tan B. (2023). Covalent triazine frameworks (CTFs): synthesis, crystallization, and photocatalytic water splitting. Chem.–Eur. J..

[cit9] Sun T., Liang Y., Luo W., Zhang L., Cao X., Xu Y. (2022). a general strategy for kilogram-scale preparation of highly crystalline covalent triazine frameworks. Angew. Chem..

[cit10] Han X., Zhao F., Shang Q., Zhao J., Zhong X., Zhang J. (2022). effect of nitrogen atom introduction on the photocatalytic hydrogen evolution activity of covalent triazine frameworks: experimental and theoretical study. ChemSusChem.

[cit11] He W., Zhou J., Xu W., Li C., Li J., Wang N. (2024). regulating the content of donor unit in donor–acceptor covalent triazine frameworks for promoting photocatalytic H_2_ production. ChemSusChem.

[cit12] Sun R., Tan B. (2022). covalent triazine frameworks (CTFs) for photocatalytic applications. Chem. Res. Chin. Univ..

[cit13] Liu M., Guo L., Jin S., Tan B. (2019). covalent triazine frameworks: synthesis and applications. J. Mater. Chem. A.

[cit14] Shen R., Li N., Qin C., Li X., Zhang P., Li X., Tang J. (2023). heteroatom- and bonded z-scheme channels-modulated ultrafast carrier dynamics and exciton dissociation in covalent triazine frameworks for efficient photocatalytic hydrogen evolution. Adv. Funct. Mater..

[cit15] Shanavaz H., Prasanna B. P., Archana S., Prashanth M. K., Alharthi F. A., Zhou R., Raghu M. S., Jeon B. H., Kumar K. Y. (2023). niobium doped triazine based covalent organic frameworks for supercapacitor applications. J. Energy Storage.

[cit16] Wu B., Liu Y., Zhang Y., Fan L., Li Q. Y., Yu Z., Zhao X., Zheng Y. C., Wang X. J. (2022). molecular engineering of covalent triazine frameworks for highly enhanced photocatalytic aerobic oxidation of sulfides. J. Mater. Chem. A.

[cit17] Liu Y., Wu H., Wang Q. (2023). strategies to improve the photocatalytic performance of covalent triazine frameworks. J. Mater. Chem. A.

[cit18] Liao L., Li M., Yin Y., Chen J., Zhong Q., Du R., Liu S., He Y., Fu W., Zeng F. (2023). advances in the synthesis of covalent triazine frameworks. ACS Omega.

[cit19] Lan Z. A., Wu M., Fang Z., Zhang Y., Chen X., Zhang G., Wang X. (2022). ionothermal synthesis of covalent triazine frameworks in a NaCl-KCl-ZnCl_2_ eutectic salt for the hydrogen evolution reaction. Angew. Chem., Int. Ed..

[cit20] Gorbunov V., Buryak A., Oskolok K., Popov A. G., Tarkhanova I. (2023). supported ionic liquid catalysts for the oxidation of *S*-and *N*-containing compounds the effect of bronsted sites and heteropolyacid concentration. Catalysts.

[cit21] Malekghasemi S., Kariminia H. R., Plechkova N. K., Ward V. C. (2021). direct transesterification of wet microalgae to biodiesel using phosphonium carboxylate ionic liquid catalysts. Biomass Bioenergy.

[cit22] Maddaloni M., Centeno-Pedrazo A., Avanzi S., Mazumdar N. J., Manyar H., Artioli N. (2023). novel ionic liquid synthesis of bimetallic Fe–Ru catalysts for the direct hydrogenation of CO_2_ to short chain hydrocarbons. Catalysts.

[cit23] Wolny A., Chrobok A. (2022). silica-based supported ionic liquid-like phases as heterogeneous catalysts. Molecules.

[cit24] Ren C., Wang Z., Gao Q., Li J., Jiang S., Huang Q., Yang Y., Zhang J., Wang Y., Hu Y., Liu Z. (2023). novel brønsted acidic ionic liquids as high efficiency catalysts for liquid-phase beckmann rearrangement. Catalysts.

[cit25] Li Z. J., Sun J. F., Xu Q. Q., Yin J. Z. (2021). homogeneous and heterogeneous ionic liquid system: promising “ideal catalysts” for the fixation of CO_2_ into cyclic carbonates. ChemCatChem.

[cit26] Lee J. H., Jung H. I., Kim D. Y. (2020). visible light-mediated photocatalytic bromination of 2-arylimidazo [1, 2-a] pyridines using CBr_4_ as bromine source. Synth. Commun..

[cit27] Davis S. N., Whittemore D. O., Fabryka-Martin J. (1998). uses of chloride/bromide ratios in studies of potable water. Groundwater.

[cit28] Akinpelu O. I., Lawal M. M., Kumalo H. M., Mhlongo N. N. (2022). computational studies of the properties and activities of selected trisubstituted benzimidazoles as potential antitubercular drugs inhibiting MTB-FtsZ polymerization. J. Biomol. Struct. Dyn..

[cit29] Mavrova A. T., Yancheva D., Anastassova N., Anichina K., Zvezdanovic J., Djordjevic A., Markovic D., Smelcerovic A. (2015). synthesis, electronic properties, antioxidant and antibacterial activity of some new benzimidazoles. Bioorg. Med. Chem..

[cit30] Yoon Y. K., Ali M. A., Wei A. C., Shirazi A. N., Parang K., Choon T. S. (2014). benzimidazoles as new scaffold of sirtuin inhibitors: green synthesis, in vitro studies, molecular docking analysis and evaluation of their anti-cancer properties. Eur. J. Med. Chem..

[cit31] Kharazmi A., Ghorbani-Vaghei R., Kharazmi A., Azadbakht R., Koolivand M., Karakaya I., Karimi-Nami R. (2023). reduced graphene oxide/palladium nanoparticle bonded to *N, N'*-bis (2-aminophenyl)-1,2-ethanediamine: a new, highly efficient and recyclable heterogeneous catalyst for direct synthesis of 2-substituted benzimidazoles via acceptorless dehydrogenative coupling of alcohols and aromatic diamine. Res. Chem. Intermed..

[cit32] Anand S., Muthusamy A. (2017). optical, thermal and electrical properties of polybenzimidazoles derived from substituted benzimidazoles. J. Mol. Struct..

[cit33] Ramya K., Mohan R., Joseph A. (2014). interaction of benzimidazoles and benzotriazole: its corrosion protection properties on mild steel in hydrochloric acid. J. Mater.
Eng. Perform..

[cit34] Keri R. S., Adimule V., Kendrekar P., Sasidhar B. S. (2025). the nano-based catalyst for the synthesis of benzimidazoles. Top. Catal..

[cit35] Vaithiyalingam M., Mohan Kumar R., Kamaraj C., Sugumar V., Manivannan N., Kadaikunnan S., Ghodake G. (2023). facile synthesis of benzimidazoles via oxidative cyclization of acyclic monoterpene aldehyde with diamines: studies on antimicrobial and in vivo evaluation of zebrafish. Chem. Biodiversity.

[cit36] Rodenes M., Gonell F., Martín S., Corma A., Sorribes I. (2022). molecularly engineering defective basal planes in molybdenum sulfide for the direct synthesis of benzimidazoles by reductive coupling of dinitroarenes with aldehydes. JACS Au.

[cit37] Koyanagi A., Murata Y., Hayakawa S., Matsumura M., Yasuike S. (2022). one-pot synthesis of 2-arylated and 2-alkylated benzoxazoles and benzimidazoles based on triphenylbismuth dichloride-promoted desulfurization of thioamides. Beilstein J. Org. Chem..

[cit38] Mahire V. N., Mahulikar P. P. (2015). facile one-pot clean synthesis of benzimidazole motifs: Exploration on bismuth nitrate accelerated subtle catalysis. Chin. Chem. Lett..

[cit39] Kusuma S., Bawiskar D. B., Singh C., Panneerselvam P., Sinha P., Samal A. K., Jadhav A. H. (2023). facile one pot synthesis of 2-substituted benzimidazole derivatives under mild conditions by using engineered MgO@DFNS as heterogeneous catalyst. RSC Adv..

[cit40] Li H., Wu H., Zhang H., Su Y., Yang S., Hensen E. J. (2019). a facile direct route to *N*-(Un) substituted lactams by cycloamination of oxocarboxylic acids without external hydrogen. ChemSusChem.

[cit41] Wu H., Yu Z., Li Y., Xu Y., Li H., Yang S. (2020). hot water-promoted catalyst-free reductive cycloamination of (bio-) keto acids with HCOONH_4_ toward cyclic amides. J. Supercrit. Fluids.

[cit42] Wu H., Dai W., Saravanamurugan S., Li H., Yang S. (2019). quasi-catalytic approach to *N*-unprotected lactams via transfer hydro-amination/cyclization of biobased keto acids. ACS Sustainable Chem. Eng..

[cit43] Kharazmi A., Ghorbani-Vaghei R., Khazaei A., Karakaya I., Karimi-Nami R. (2025). application of novel silica stabilized on a covalent triazine framework as a highly efficient heterogeneous and recyclable catalyst in the effective green synthesis of porphyrins. RSC Adv..

[cit44] Hajjami M., Ghorbani-Choghamarani A., Norouzi M. (2012). an efficient and facile procedure for synthesis of acetates from alcohols catalyzed by poly (4-vinylpyridinium tribromide). Chin. J. Catal..

[cit45] Kharazmi A., Ghorbani-Vaghei R., Noori S., Alavinia S. (2022). synthesis of multiple quinoline derivatives using novel ionic liquid-based nano-magnetic catalyst (MNPs@SiO_2_-Pr-AP-tribromide). Res. Chem. Intermed..

[cit46] Purushothaman R., Vaitinadin H. S. (2020). inclusion of covalent triazine framework into fluorinated polyimides to obtain composites with low dielectric constant. J. Appl. Polym. Sci..

[cit47] Jin H., Zhang C., Liu P., Ge X., Zhou s. (2022). covalent organic framework-supported Pd nanoparticles: an efficient and reusable heterogeneous catalyst for Suzuki–Miyaura coupling reactions. Appl.Organomet.Chem..

[cit48] Wang M., Guo H., Wu N., Zhang J., Zhang T., Liu B., Pan Z., Peng L., Yang W. (2022). a novel triazine-based covalent organic framework combined with AuNPs and reduced graphene oxide as an electrochemical sensing platform for the simultaneous detection of uric acid, dopamine and ascorbic acid. Colloids Surf., A.

[cit49] Liu Q., Yang S., Repich H., Zhai Y., Xu X., Liang Y., Li H., Wang H., Xu F. (2020). porous functionalized covalent-triazine frameworks for enhanced adsorption toward polysulfides in Li-S batteries and organic dyes. Front. Chem..

[cit50] Nandi D. K., Seth J., Chakrabortty P., Ghosh S., Chowdhury A., Bhaumik A., Islam S. M. (2023). Ni nanoparticles supported over triazine based porous organic polymer for selective CO_2_ photo-reduction to methanol. ChemCatChem.

[cit51] Mayer U., Gerger W., Gutmann V. (1977). NMR-spectroscopic studies on solvent electrophilic properties, Part II: Binary aqueous-non aqueous solvent systems. Monatsh. Chem..

[cit52] Hanoon H. D., Kowsari E., Abdouss M., Zandi H., Ghasemi M. H. (2017). efficient preparation of acidic ionic liquid-functionalized reduced graphene oxide and its catalytic performance in synthesis of benzimidazole derivatives. Res. Chem. Intermed..

[cit53] Srinivasulu R., Kumar K. R., Satyanarayana P. V. V. (2014). facile and efficient method for synthesis of benzimidazole derivatives catalyzed by zinc triflate. Green Sustainable Chem..

[cit54] Inamdar S. M., More V. K., Mandal S. K. (2013). CuO nano-particles supported on silica, a new catalyst for facile synthesis of benzimidazoles, benzothiazoles and benzoxazoles. Tetrahedron Lett..

[cit55] Yang Q., Yin Z. L., Ouyang B. L., Peng Y. Y. (2011). Pyridinium tribromide catalyzed condensation of indoles and aldehydes to form bisindolylalkanes. Chin. Chem. Lett..

