# Supplementary material for: Design of a novel tribromide ionic liquid covalent triazine framework for green synthesis of benzimidazoles
Source: RSC Adv. 2026 Jul 3;16(35):36177–92. doi: 10.1039/d6ra04048a (PMC13330587; doi:10.1039/d6ra04048a)
Supplement: RA-016-D6RA04048A-s001 [file RA-016-D6RA04048A-s001.pdf]

Supplementary Information (SI) for RSC Advances.
This journal is © The Royal Society of Chemistry 2026

Supporting Information

Design of a Novel Tribromide Ionic Liquid Covalent Triazine Framework for Green Synthesis of Benzimidazoles

Azin Kharazmi,[a] Ramin Ghorbani-Vaghei,*[,a,b] Ardeshir Khazaei,[a] Idris Karakaya,[c] and Rahman Karimi-Nami[d]

[a]*Department of Organic Chemistry, Faculty of Chemistry and Petroleum Sciences, Bu-Ali Sina University, Hamedan 6517838683, Iran.*

[b]*Department of Organic Chemistry, Faculty of Chemistry,University of Guilan, Rasht, Iran*

[c]*Department of Chemistry, Colleage of Basic Sciences, Gebze Technical University, 41400 Gebze, Türkiye*

[d]*Department of Chemistry, Faculty of Science, University of Maragheh, P.O Box 55181-83111, Maragheh, Iran.*

**Corresponding author: Phone: +989183122123; Fax: +988138380709; E-mail address:* rgvaghei@yahoo.com & *A.RGV@guilan.ac.ir*

Contents:

Analytical data and Copies of ^{1}HNMR and ^{13}CNMR spectra:

Figure S1. ^{1}HNMR spectra of *N^2,N^4,N^6*-tris(2-aminophenyl)-1,3,5-triazine-2,4,6-triamine S1

Figure S2. ^{13}CNMR spectra of *N^2,N^4,N^6*-tris(2-aminophenyl)-1,3,5-triazine-2,4,6-triamine S2

Figure S3. Mass spectra of *N^2,N^4,N^6*-tris(2-aminophenyl)-1,3,5-triazine-2,4,6-triamine S3

Figure S4. ^{1}HNMR spectra of 4,4'-(butane-1,4-diyl*bis*(oxy))dibenzaldehyde S4

Figure S5. ^{13}CNMR spectra of 4,4'-(butane-1,4-diyl*bis*(oxy))dibenzaldehyde S5

Figure S6. Mass spectra of 4,4'-(butane-1,4-diyl*bis*(oxy))dibenzaldehyde S6

Figure S7. ^{1}HNMR spectra of 2-(4-fluorophenyl)-1*H*-benzo[*d*]imidazole S7

Figure S8. ^{13}CNMR spectra of 2-(4-fluorophenyl)-1*H*-benzo[*d*]imidazole S8

Figure S9. ^{1}HNMR spectra of 2-(thiophen-2-yl)-1*H*-benzo[*d*]imidazole S9

Figure S10. ^{13}CNMR spectra of 2-(thiophen-2-yl)-1*H*-benzo[*d*]imidazole S10

Figure S11. ^{1}HNMR spectra of 1,2-*bis*(1*H*-benzo[*d*]imidazol-2-yl)benzene S11

Figure S12. ^{13}CNMR spectra of 1,2-*bis*(1*H*-benzo[*d*]imidazol-2-yl)benzene S12

Figure S13. ^{1}HNMR spectra of 1,4-*bis*(1*H*-benzo[*d*]imidazol-2-yl)benzene S13

Figure S14. ^{13}CNMR spectra of 1,4-*bis*(1*H*-benzo[*d*]imidazol-2-yl)benzene S14

Figure S15. ^{1}HNMR spectra of 1,4-*bis*(4-(1*H*-benzo[*d*]imidazol-2-yl)phenoxy)butane S15

Figure S16. ^{13}CNMR spectra of 1,4-*bis*(4-(1*H*-benzo[*d*]imidazol-2-yl)phenoxy)butane S16

Figure S17. 1HNMR spectra of 2-(naphthalen-1-yl)-1*H*-benzo[*d*]imidazole S17

Figure S18. ^{13}**C**NMR spectra of 2-(naphthalen-1-yl)-1*H*-benzo[*d*]imidazole S18

Figure S19. ^{1}HNMR spectra of 2-(6-bromonaphthalen-1-yl)-1*H*-benzo[*d*]imidazole S19

Figure S20. ^{13}CNMR spectra of 2-(6-bromonaphthalen-1-yl)-1*H*-benzo[*d*]imidazole S20

Figure S21. ^{1}HNMR spectra of 2-(anthracen-1-yl)-1*H*-benzo[*d*]imidazole S21

Figure S22. ^{13}CNMR spectra of 2-(anthracen-1-yl)-1*H*-benzo[*d*]imidazole S22

Figure S23. ^{1}HNMR spectra of 4-(1*H*-benzo[*d*]imidazol-2-yl)phenyl 4-methylbenzenesulfonate S23

Figure S24. ^{13}CNMR spectra of 4-(1*H*-benzo[*d*]imidazol-2-yl)phenyl 4-methylbenzenesulfonate S24

2. Analytical data and Copies of ¹HNMR and ¹³CNMR spectra:

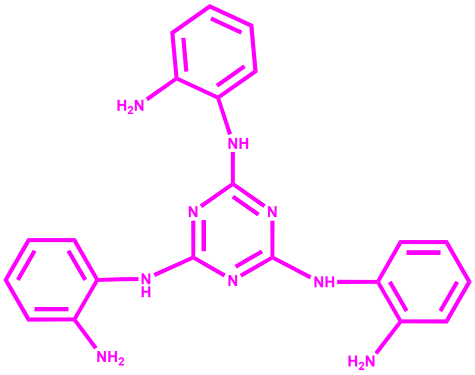

^{1}H NMR (400 MHz, DMSO-d_6) δ 8.10 (s, 2H), 7.32 (t, J = 3.6 Hz, 3H), 7.23 (d, J = 2.1 Hz, 3H), 6.70 (t, J = 3.4 Hz, 8H), 6.49 (d, J = 5.3 Hz, 3H), 4.91 (s, 6H). ^{13}C NMR (101 MHz, DMSO) δ 163.88, 142.49, 128.52, 127.14, 126.94, 116.63, 116.25.

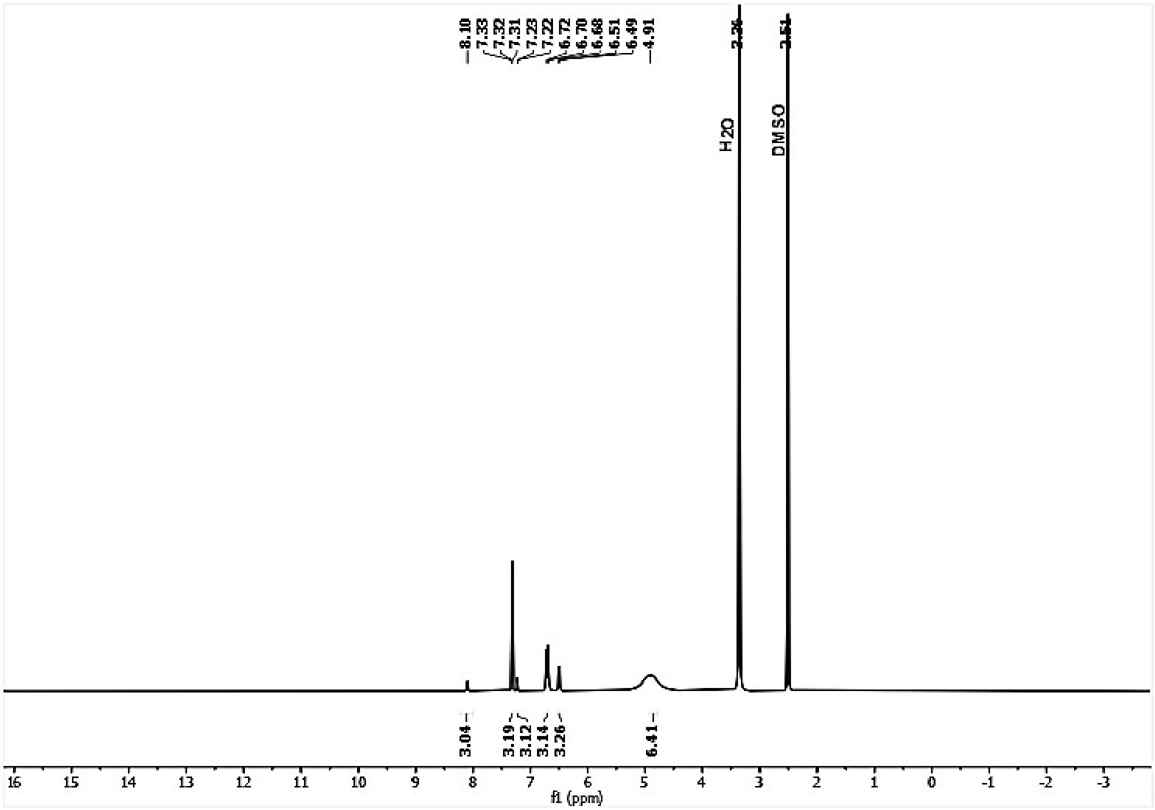

Figure S1. ¹HNMR spectra of *N²,N⁴,N⁶*-tris(2-aminophenyl)-1,3,5-triazine-2,4,6-triamine.

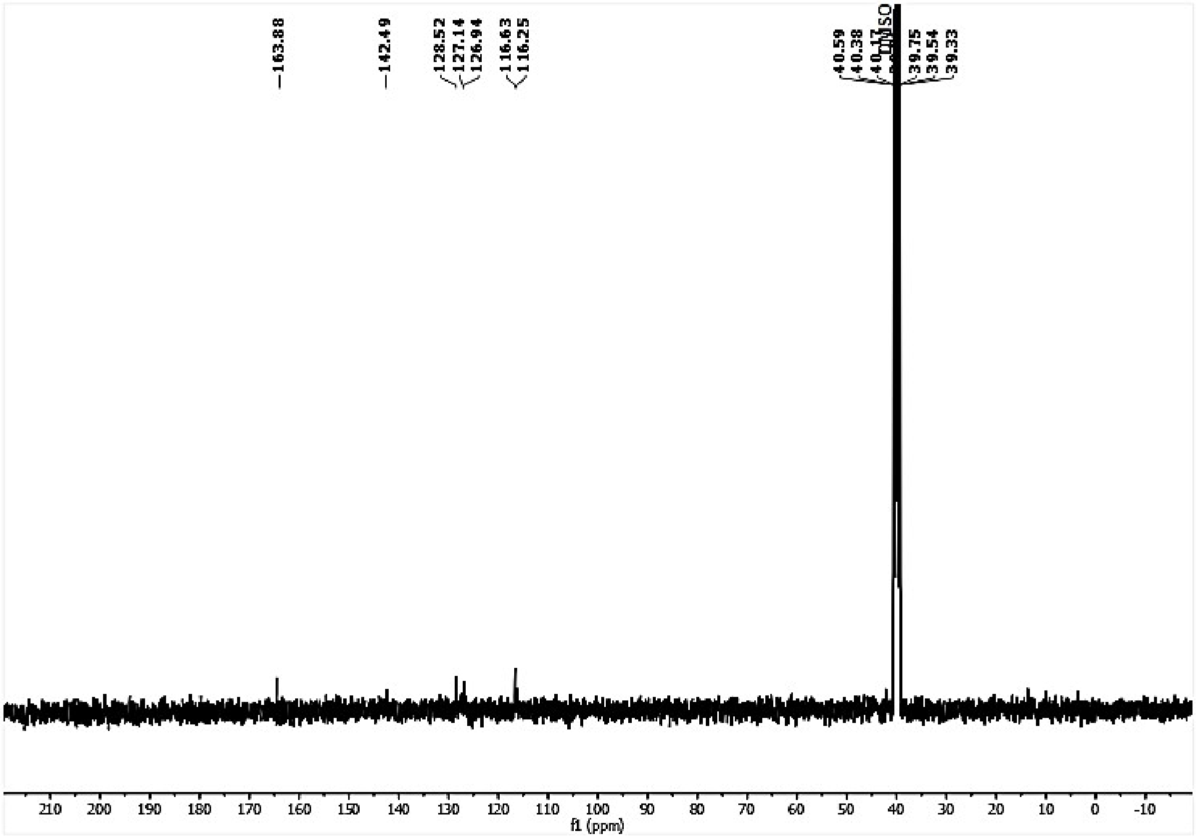

Figure S2. ^{13}CNMR spectra of N^2,N^4,N^6-tris(2-aminophenyl)-1,3,5-triazine-2,4,6-triamine.

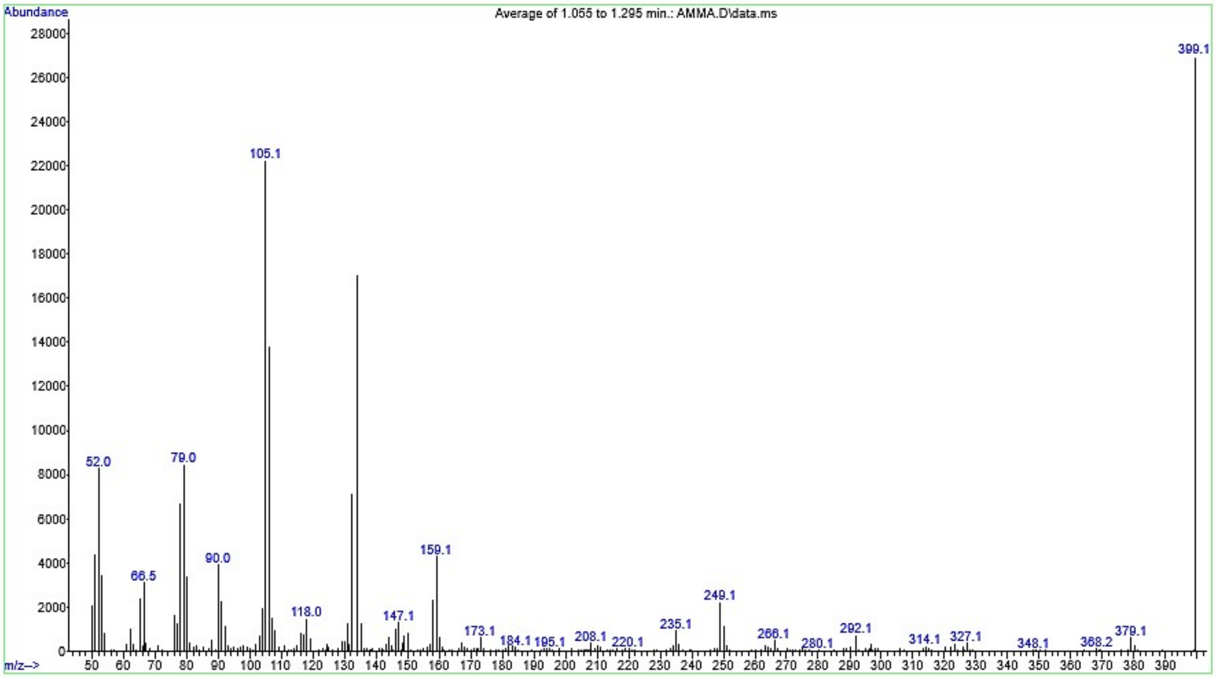

Figure S3. Mass spectra of N^2,N^4,N^6-tris(2-aminophenyl)-1,3,5-triazine-2,4,6-triamine.

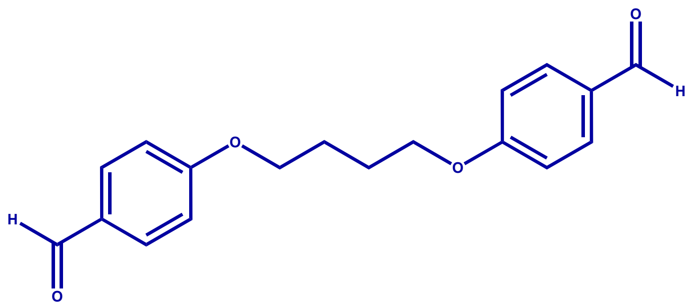

^{1}H NMR (400 MHz, DMSO-d_6) δ 9.87 (s, 2H), 7.87 (d, *J* = 8.7 Hz, 4H), 7.14 (d, *J* = 8.7 Hz, 4H), 4.17 (t, *J* = 5.6 Hz, 4H), 1.92 (q, *J* = 3.0 Hz, 4H). ^{13}C NMR (101 MHz, DMSO) δ 191.78, 164.07, 132.30, 130.02, 115.39, 68.12, 25.61.

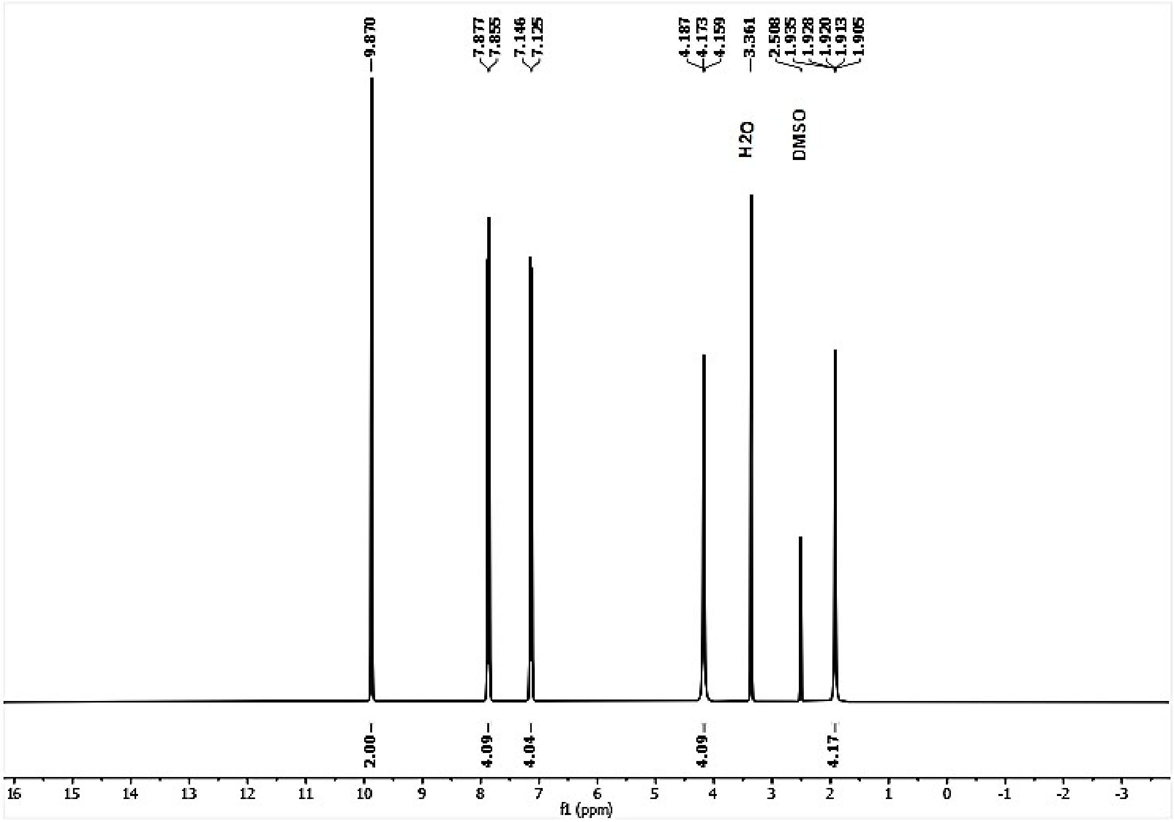

Figure S4. ^{1}HNMR spectra of 4,4'-(butane-1,4-diyl*bis*(oxy))dibenzaldehyde.

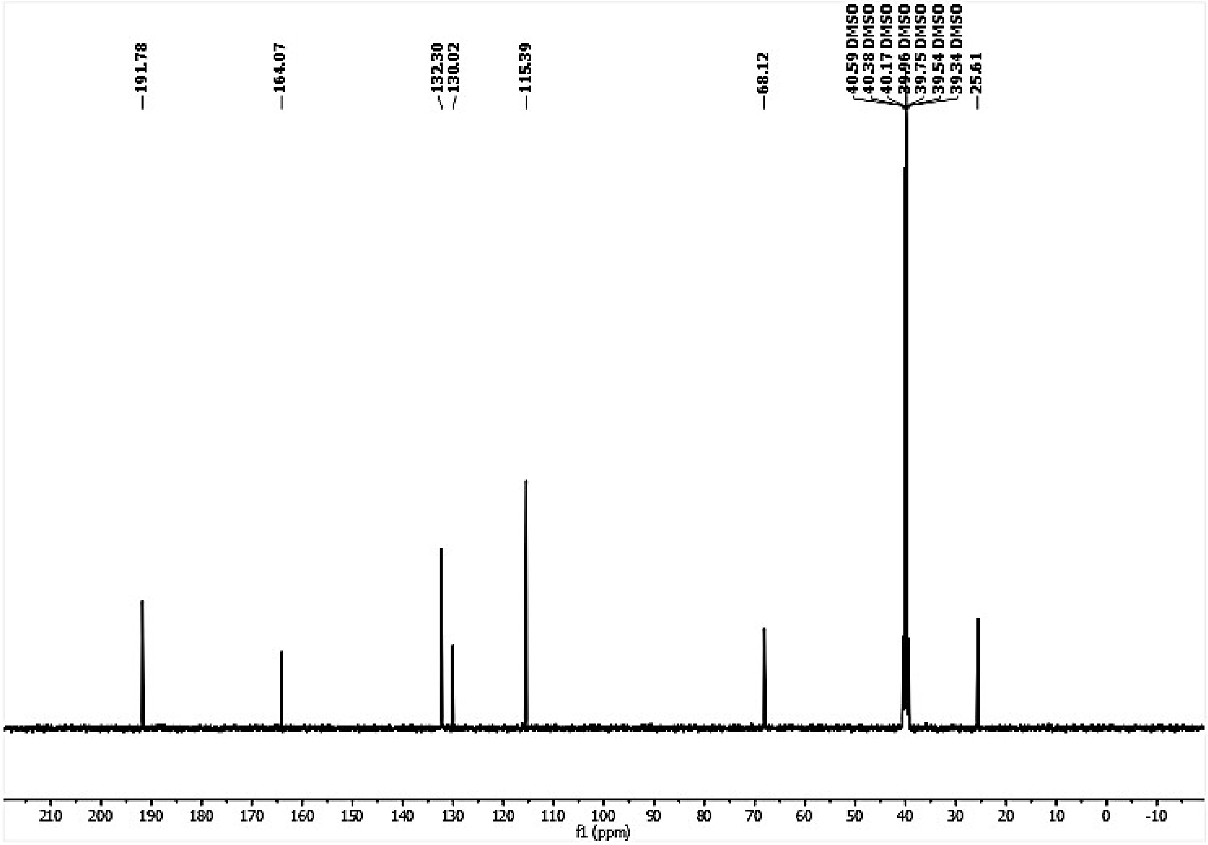

Figure S5. ^{13}CNMR spectra of 4,4'-(butane-1,4-diyl*bis*(oxy))dibenzaldehyde.

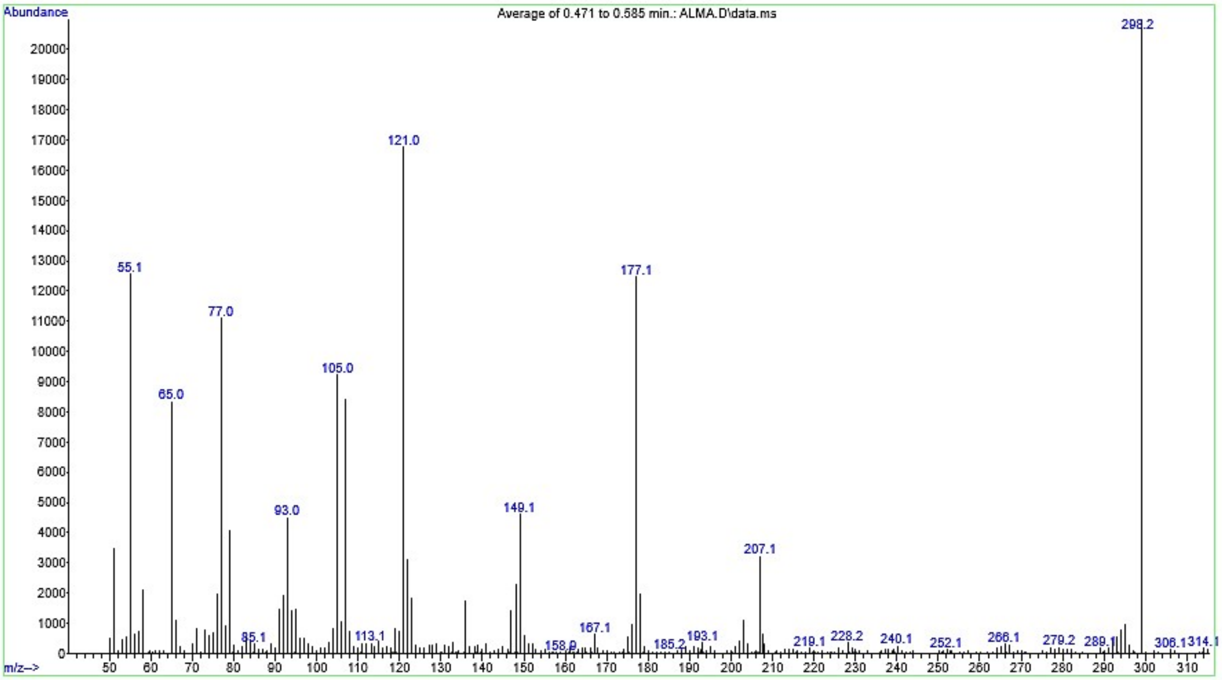

Figure S6. Mass spectra of 4,4'-(butane-1,4-diyl*bis*(oxy))dibenzaldehyde.

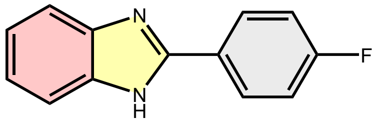

Compound 3a: 98% yield; ^{1}H NMR (400 MHz, DMSO-d_6) δ 12.96 (s, 1H), 8.35 – 8.13 (m, 4H), 7.40 (d, J = 8.9 Hz, 2H), 7.21 (t, J = 6.0 Hz, 2H). ^{13}C NMR (101 MHz, DMSO) δ 162.30, 150.84, 141.27, 129.24, 129.15, 122.62, 116.61, 116.39.

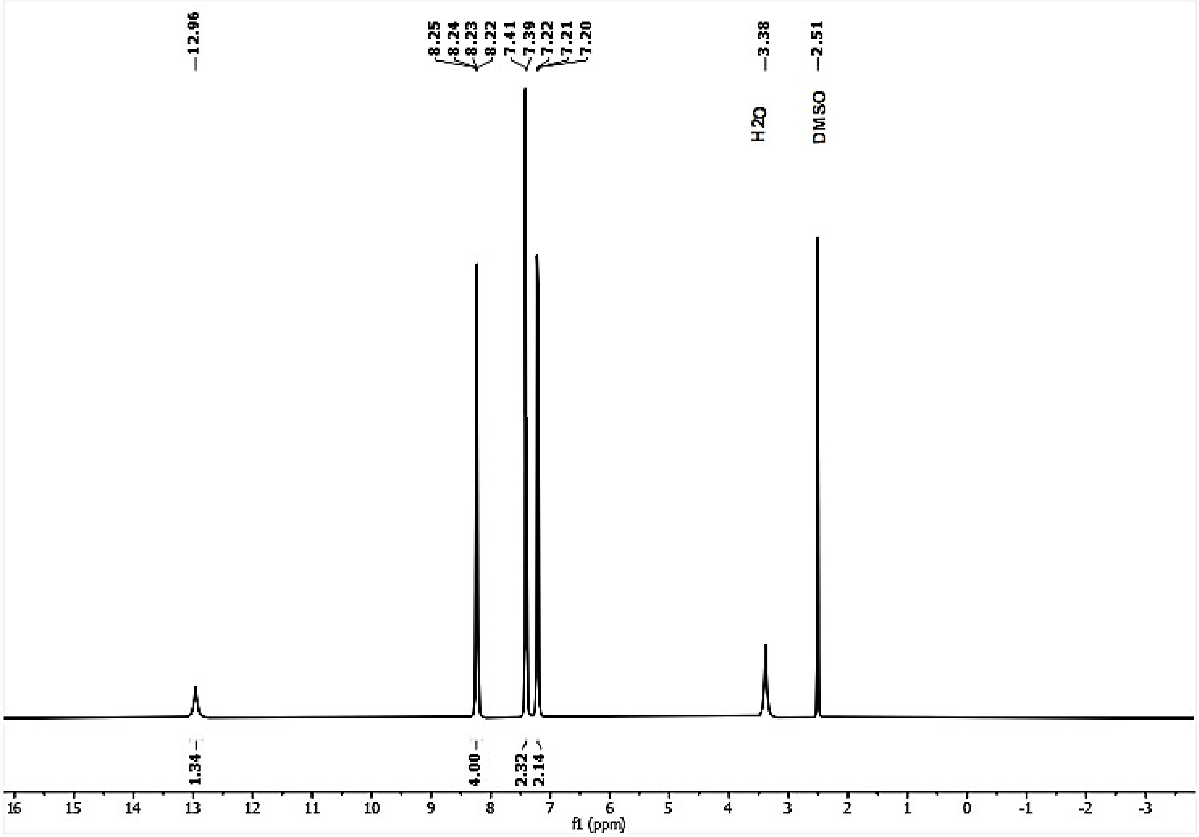

Figure S7. ^{1}HNMR spectra of 2-(4-fluorophenyl)-1H-benzo[d]imidazole (compound 3a).

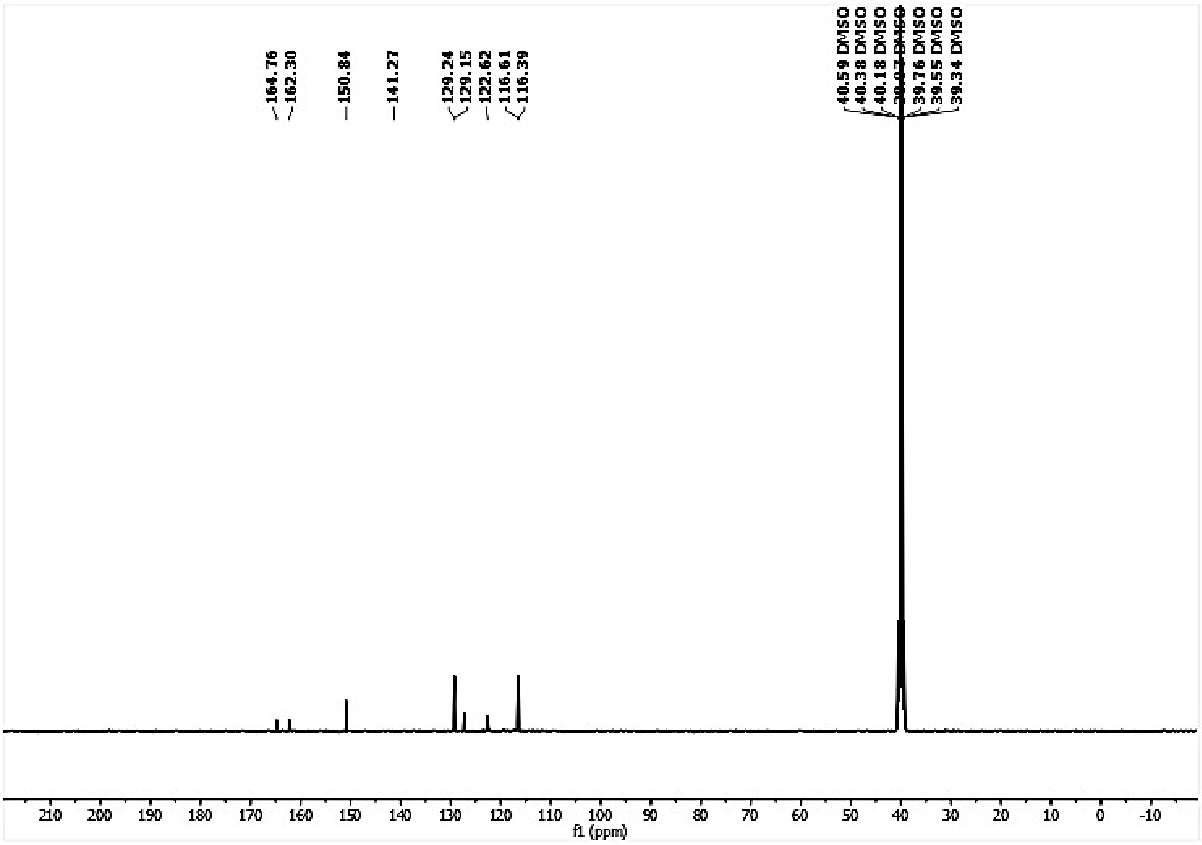

Figure S8. ^{13}CNMR spectra of 2-(4-fluorophenyl)-1*H*-benzo[*d*]imidazole (compound 3a).

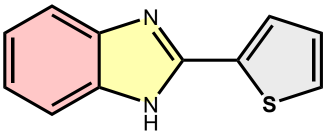

Compound 3b: 90% yield; ^{1}H NMR (400 MHz, DMSO-d_6) δ 12.99 (s, 1H), 7.85 (d, J = 3.7 Hz, 1H), 7.73 (d, J = 5.0 Hz, 1H), 7.56 (t, J = 4.1Hz, 2H), 7.27 – 7.16 (m, 3H). ^{13}C NMR (101 MHz, DMSO) δ 147.49, 142.53, 141.27, 134.16, 129.25, 128.77, 122.67, 115.43.

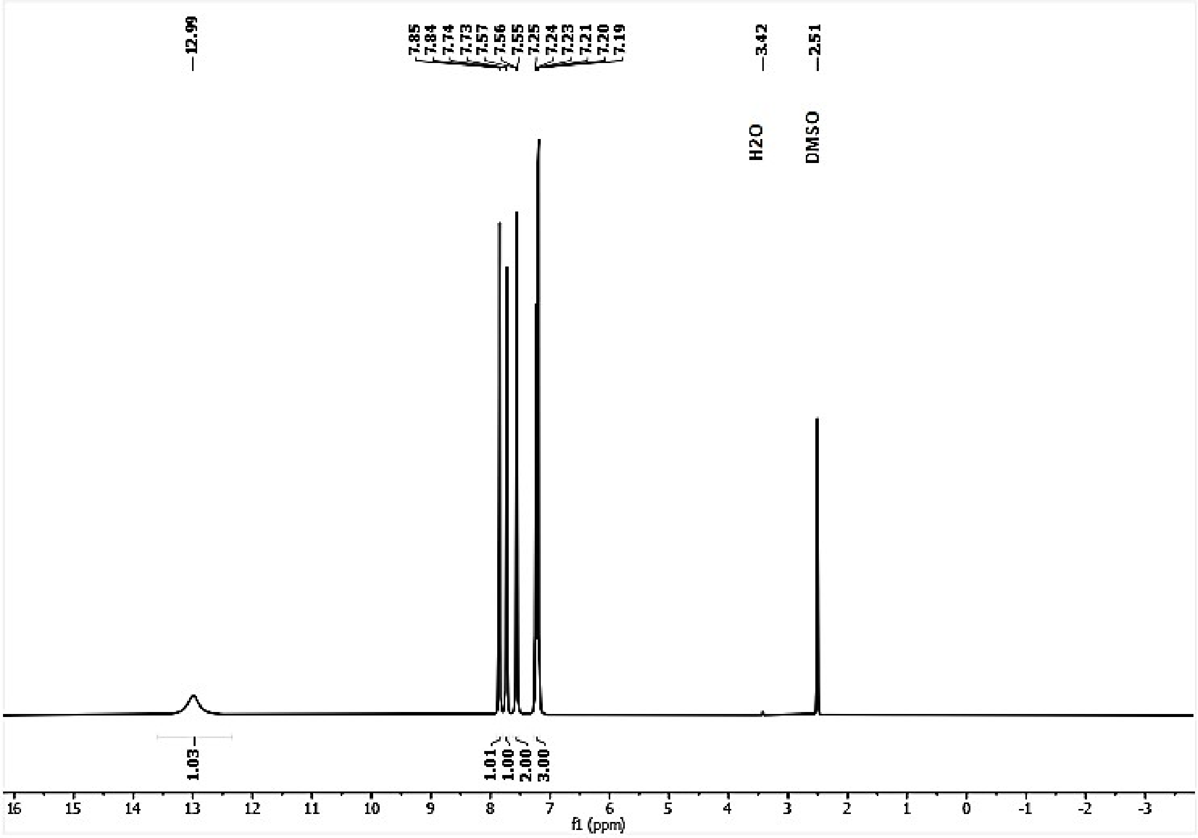

Figure S9. ^{1}HNMR spectra of 2-(thiophen-2-yl)-1H-benzo[d]imidazole (compound 3b).

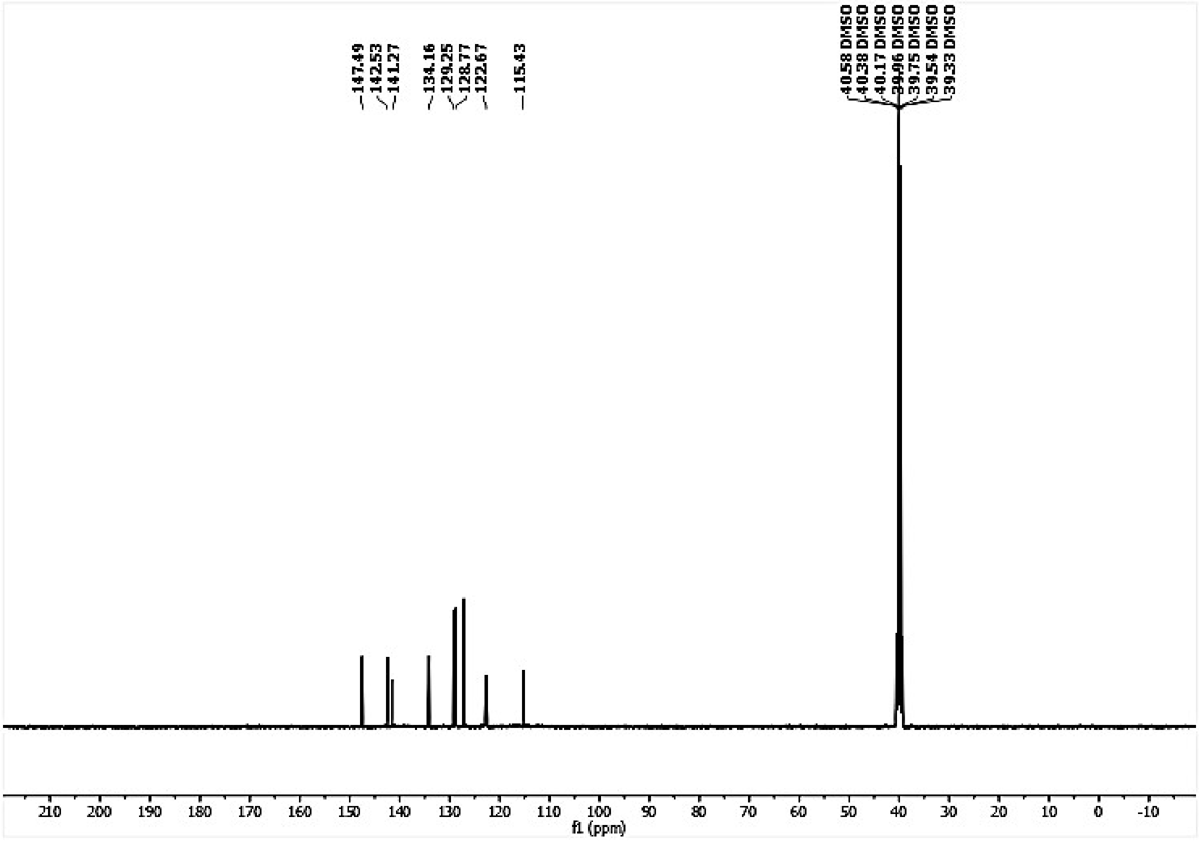

Figure S10. ^{13}CNMR spectra of 2-(thiophen-2-yl)-1*H*-benzo[*d*]imidazole (compound 3b).

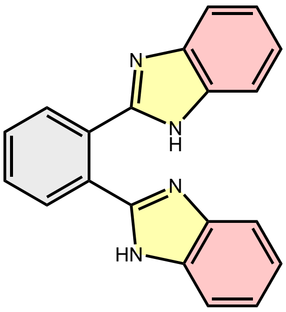

Compound 3c: 60% yield; ^{1}H NMR (250 MHz, DMSO-d_6) δ ^{1}H NMR (250 MHz, DMSO-d_6) δ ^{1}H NMR (250 MHz, DMSO-d_6) δ 12.61 (s, 1H), 7.93 (d, J = 5.0 Hz, 2H), 7.68 (d, J = 7.5 Hz, 4H), 7.55 (t, J = 6.3 Hz, 2H), 7.24 (t, J = 6.9 Hz, 4H). ^{13}C NMR (63 MHz, DMSO) δ 158.61, 145.05, 130.02, 128.92, 125.08, 122.62, 120.20.

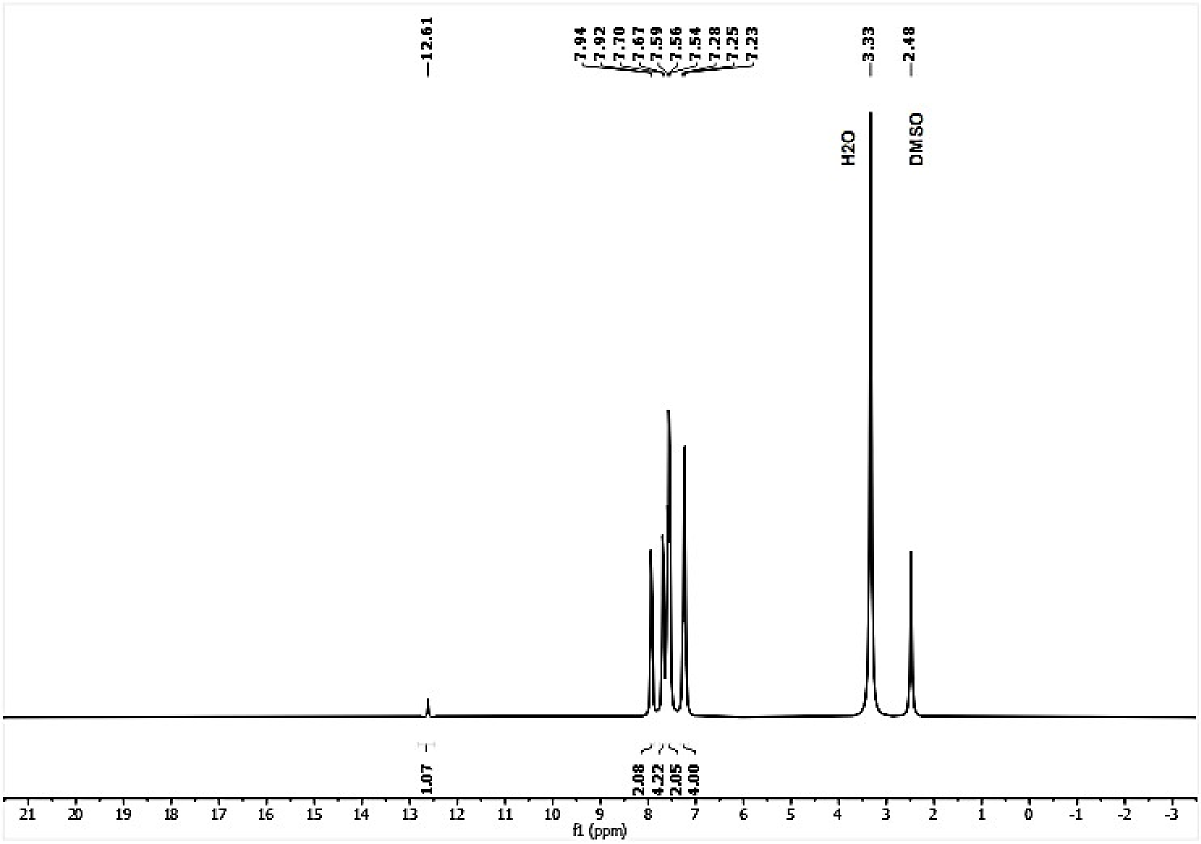

Figure S11. ^{1}HNMR spectra of 1,2-*bis*(1*H*-benzo[*d*]imidazol-2-yl)benzene (compound 3c).

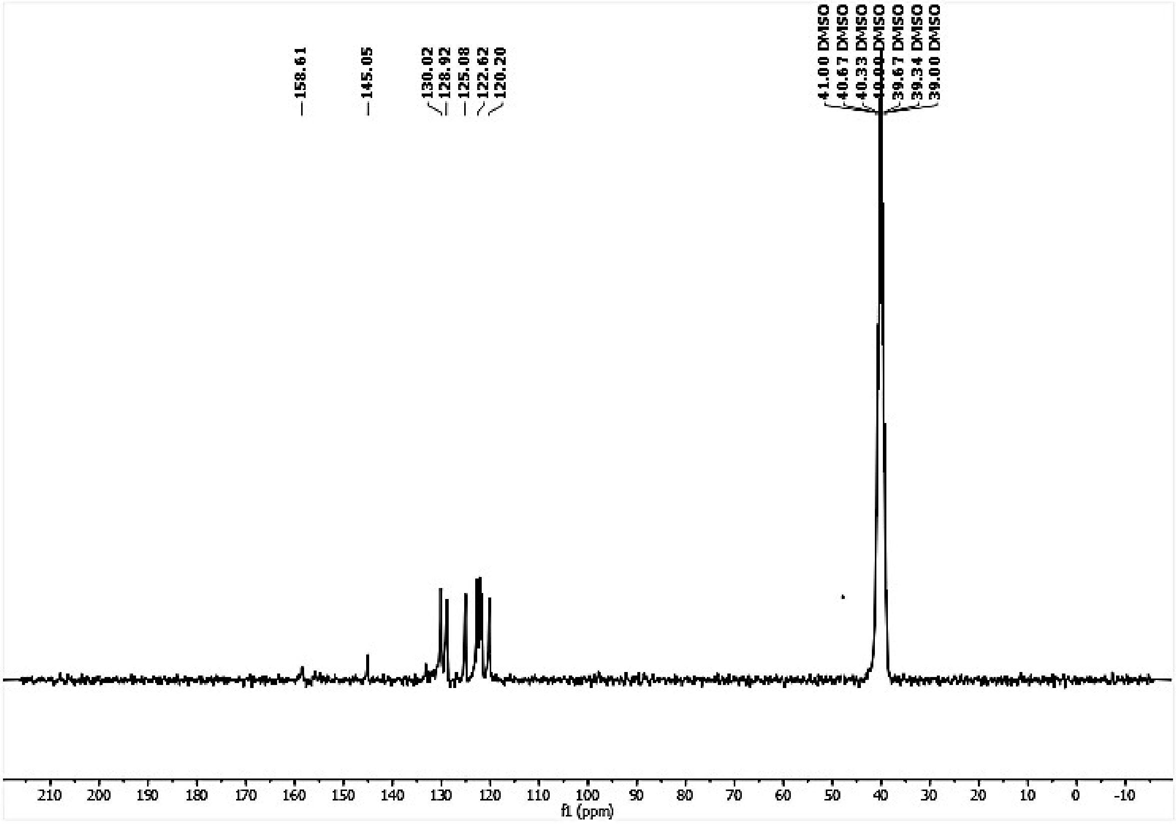

Figure S12. ^{13}CNMR spectra of 1,2-*bis*(1*H*-benzo[*d*]imidazol-2-yl)benzene (compound 3c).

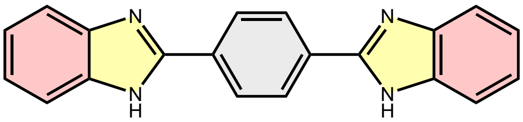

Compound 3d: 95% yield; ^{1}H NMR (400 MHz, DMSO-d_6) δ 13.14 (s, 2H), 8.36 (s, 4H), 7.64 (d, J = 6.0 Hz, 4H), 7.25 (t, J = 4.0 Hz, 4H). ^{13}C NMR (101 MHz, DMSO) δ 150.95, 140.09, 131.48, 127.42, 122.93, 116.53. ^{13}C NMR (101 MHz, DMSO) δ 150.95, 140.09, 131.48, 127.42, 122.93, 116.53.

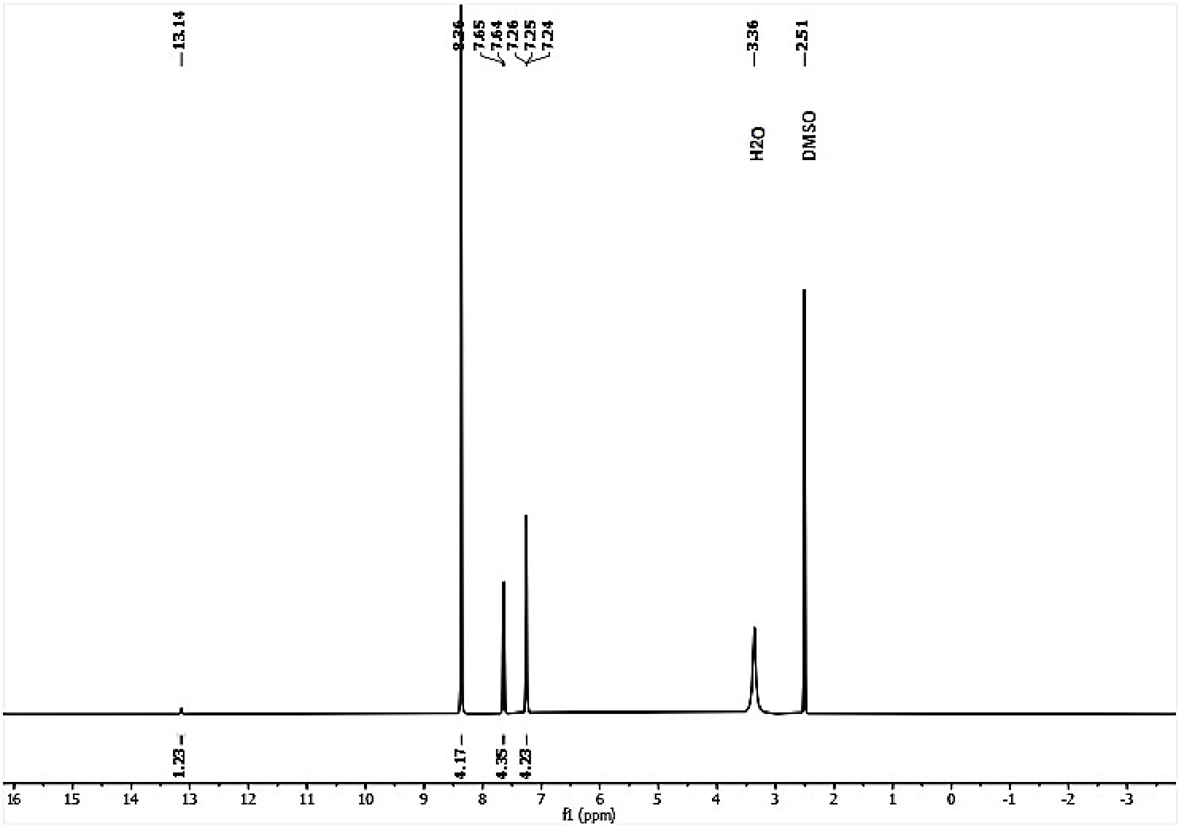

Figure S13. ^{1}HNMR spectra of 1,4-*bis*(1*H*-benzo[*d*]imidazol-2-yl)benzene (compound 3d).

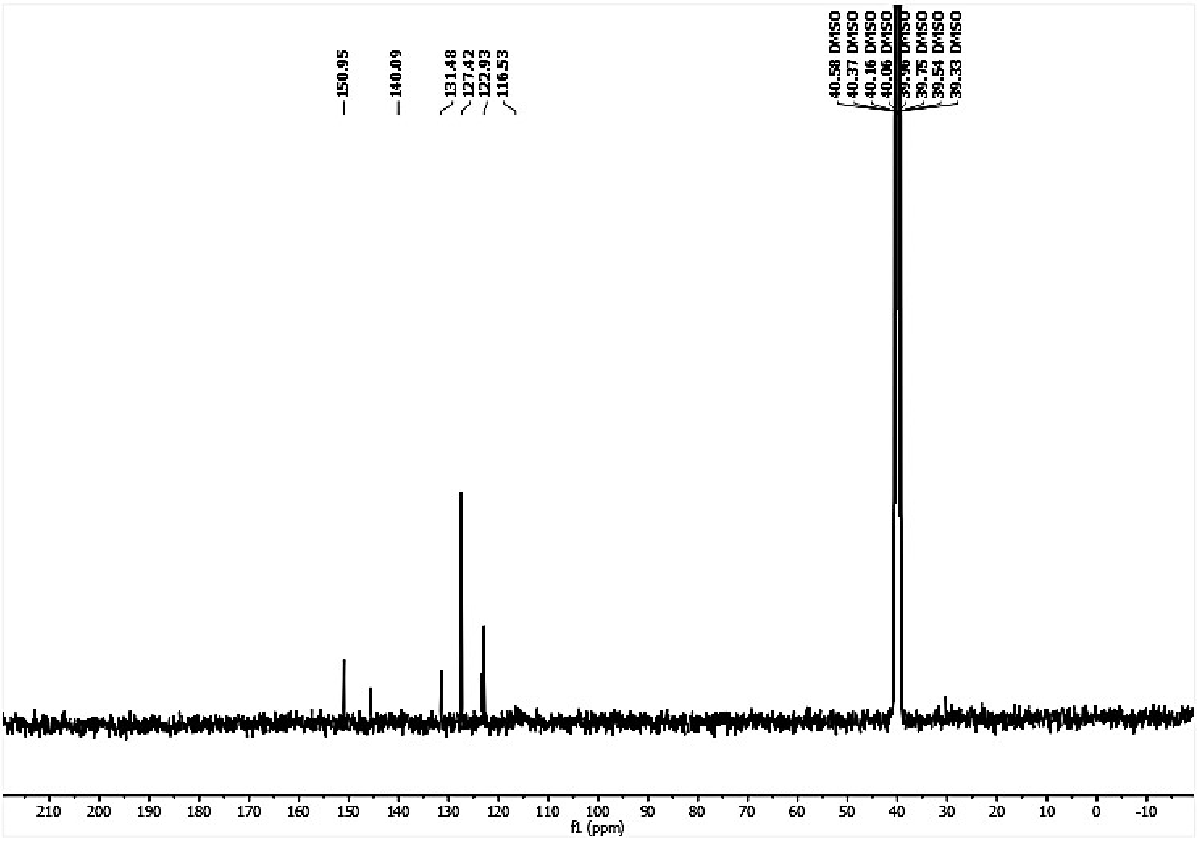

Figure S14. ^{13}CNMR spectra of 1,4-*bis*(1*H*-benzo[*d*]imidazol-2-yl)benzene (compound 3d).

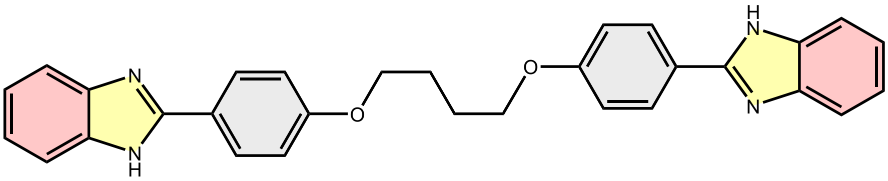

Compound 3e: 90% yield; ^{1}H NMR (400 MHz, DMSO-d_6) δ 12.56 (s, 2H), 7.68 (d, J = 8.0 Hz, 4H), 7.22 (t, J = 8.0 Hz, 4H), 7.11 (d, J = 4.0 Hz, 4H), 7.07 (d, J = 8.0 Hz, 4H), 4.10 (t, J = 8.0 Hz, 4H), 1.86 (m, 4H). ^{13}C NMR (101 MHz, DMSO) δ 159.58, 151.56, 142.62, 127.94, 122.67, 116.10, 115.14, 111.60, 67.80, 25.78.

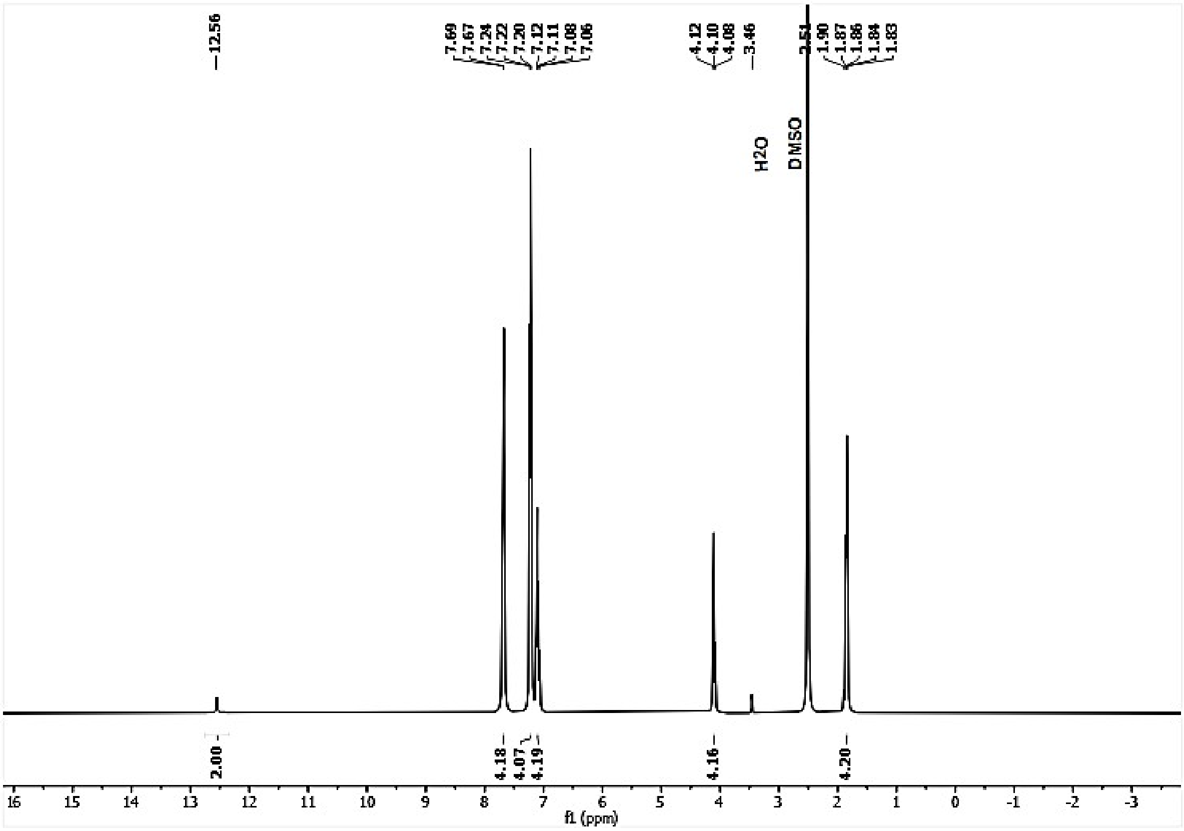

Figure S15. ^{1}HNMR spectra of 1,4-*bis*(4-(1*H*-benzo[*d*]imidazol-2-yl)phenoxy)butane (compound 3e).

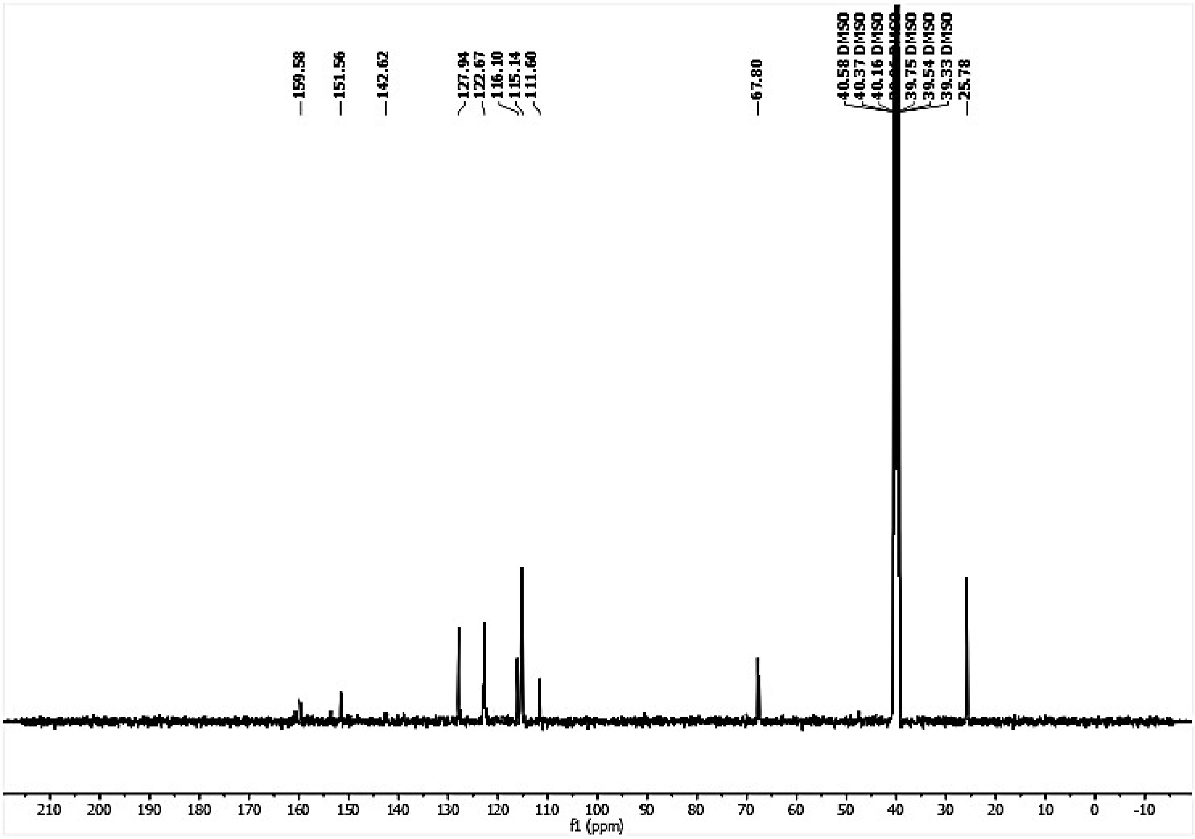

Figure S16. ^{13}CNMR spectra of 1,4-*bis*(4-(1*H*-benzo[*d*]imidazol-2-yl)phenoxy)butane (compound 3e).

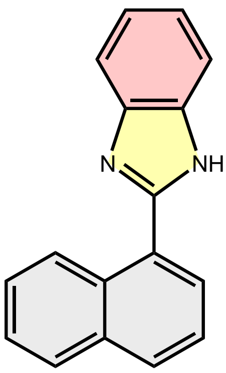

Compound 3f: 65% yield; ^{1}H NMR (250 MHz, DMSO-d_6) δ 13.03 (s, 1H), 9.10 (d, *J* = 7.5 Hz, 2H), 8.51 (m, 2H), 7.14 (d, *J* = 2.5 Hz, 1H), 8.07 (d, *J* = 7.5 Hz, 1H), 8.0 (d, *J* = 7.5 Hz, 2H), 7.29 (m, 2H), 7.24 (t, *J* = 5 Hz, 2H). ^{13}C NMR (63 MHz, DMSO) δ 151.82, 141.69, 134.07, 130.97, 130.56, 128.82, 128.32, 127.98, 127.48, 126.79, 125.72, 123.09, 122.47, 116.01.

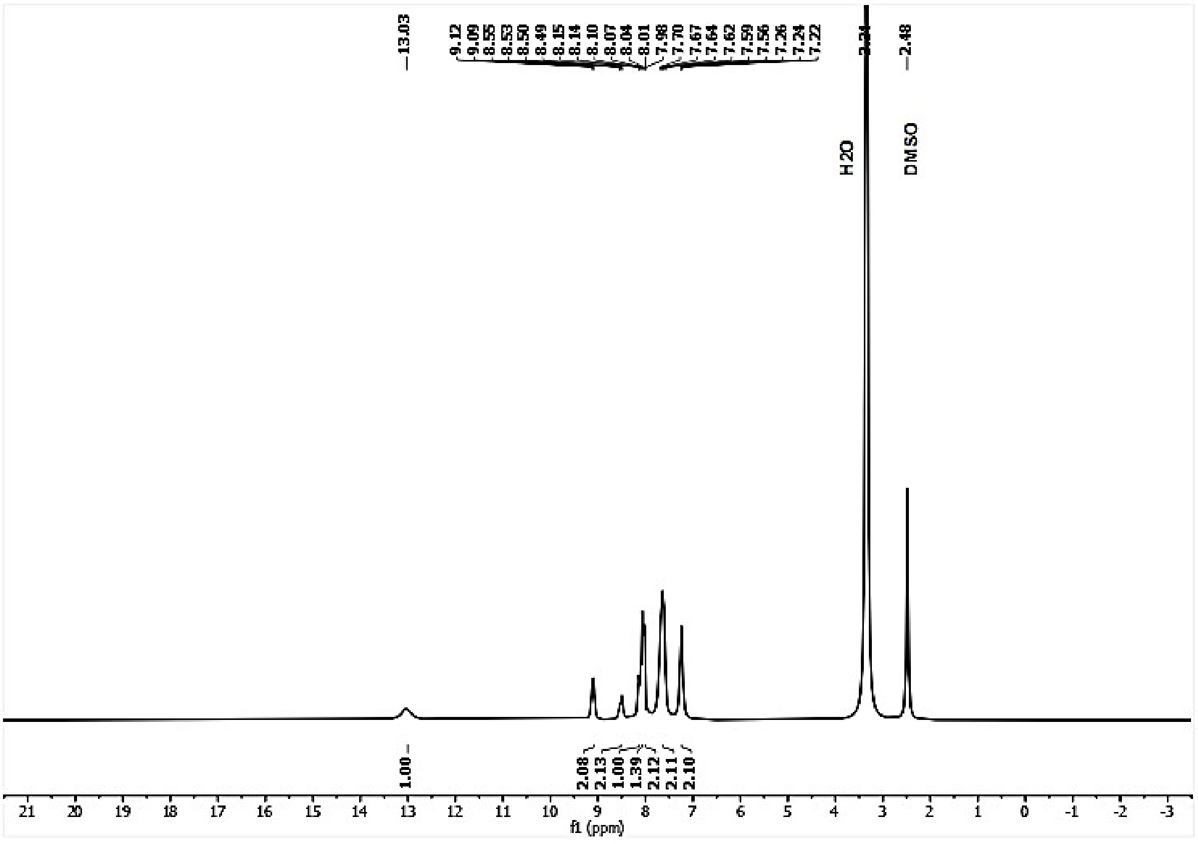

Figure S17. ^{1}HNMR spectra of 2-(naphthalen-1-yl)-1*H*-benzo[*d*]imidazole (compound 3f).

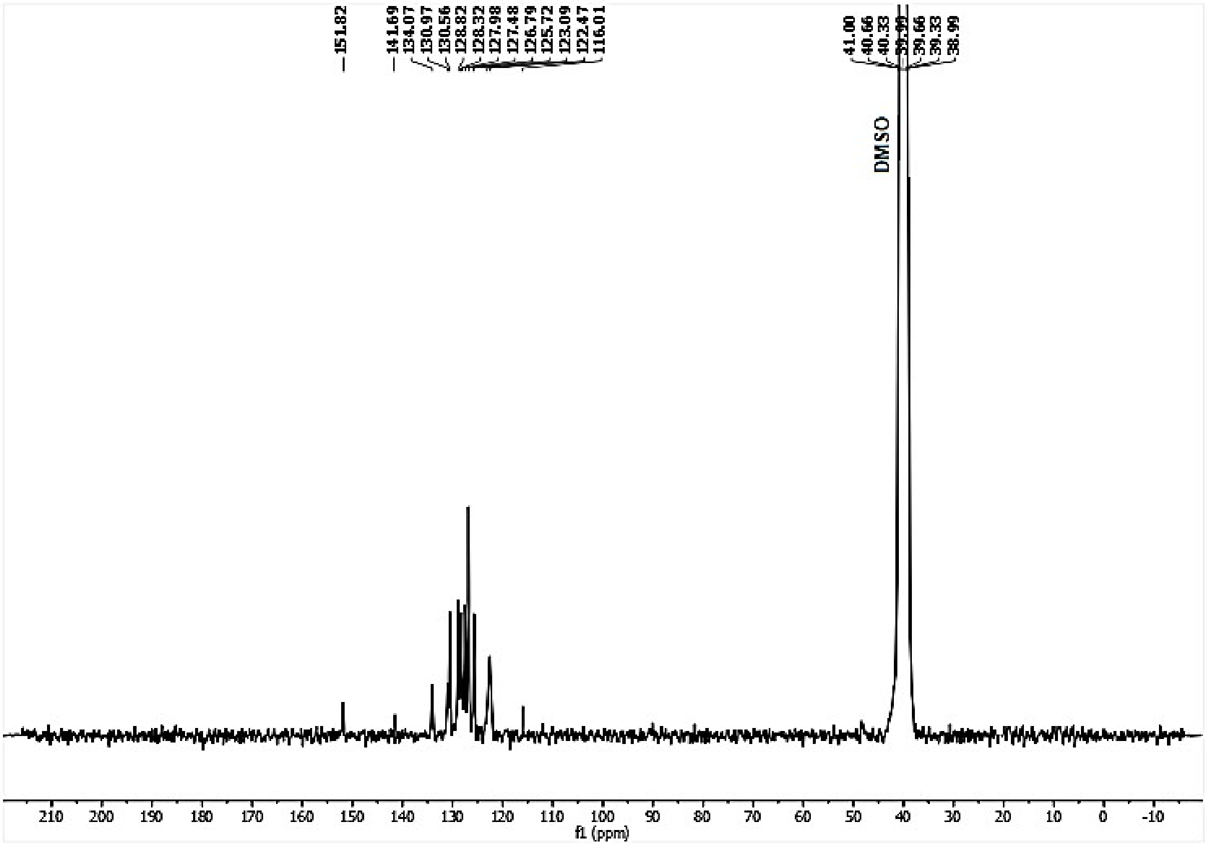

Figure S18. ^{13}CNMR spectra of 2-(naphthalen-1-yl)-1*H*-benzo[*d*]imidazole (compound 3f).

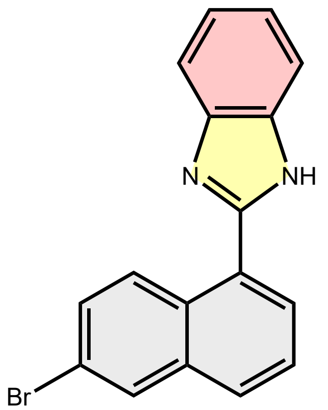

Compound 3j: 68% yield; ^{1}H NMR (250 MHz, DMSO-*d6*) δ 12.58 (s, 1H), 8.72 (s, 1H), 8.02 (t, *J*= 12.5, 1H), 8.29 (d, *J* = 8.9 Hz, 1H), 7.66 – 7.62 (m, 2H), 7.63 (d, *J*= 7.5 Hz, 2H), 7.58 (d, *J*= 7.5 Hz, 1H), 7.22 (d, *J*= 2.5, 2H). ^{13}C NMR (63 MHz, DMSO) δ 151.66, 141.64, 133.93, 133.26, 129.01, 128.89, 128.25, 127.59, 127.38, 126.31, 124.38, 122.72, 119.25, 115.38.

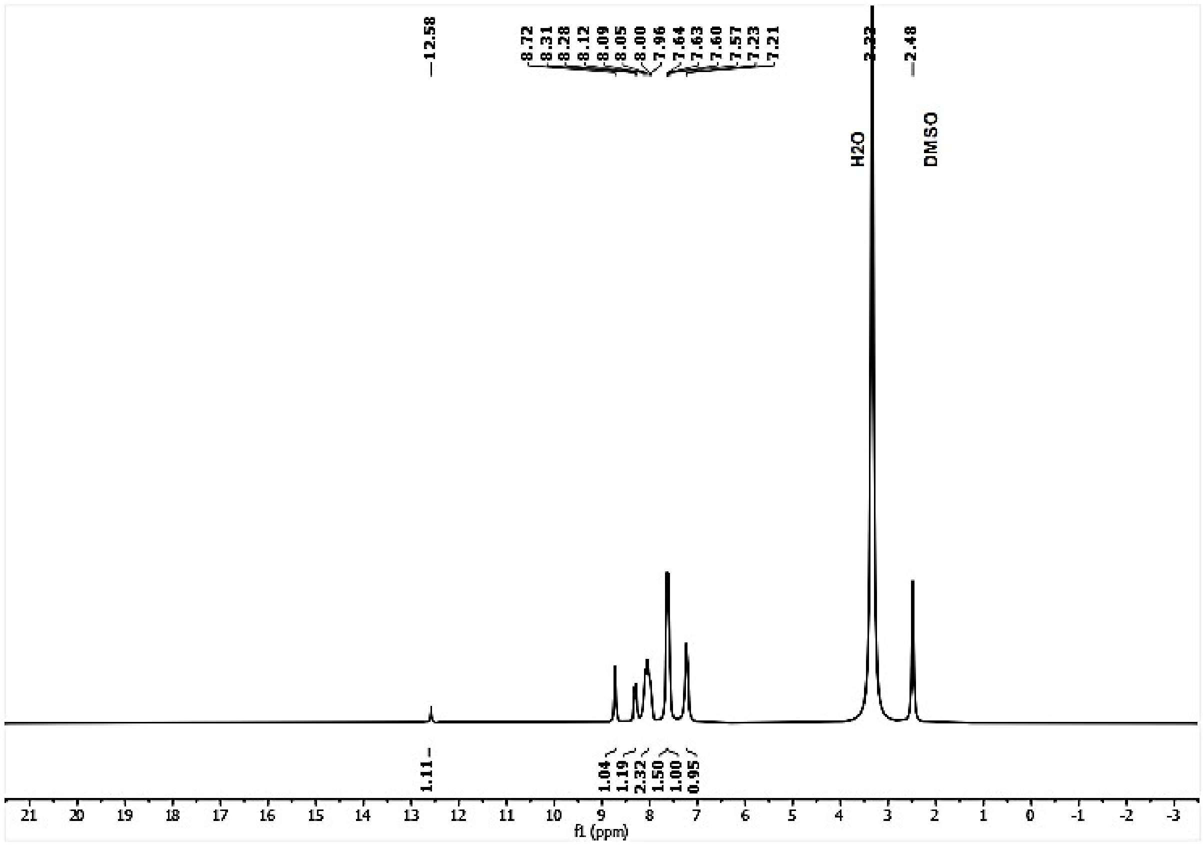

Figure S19. ^{1}HNMR spectra of 2-(6-bromonaphthalen-1-yl)-1*H*-benzo[*d*]imidazole (compound 3j).

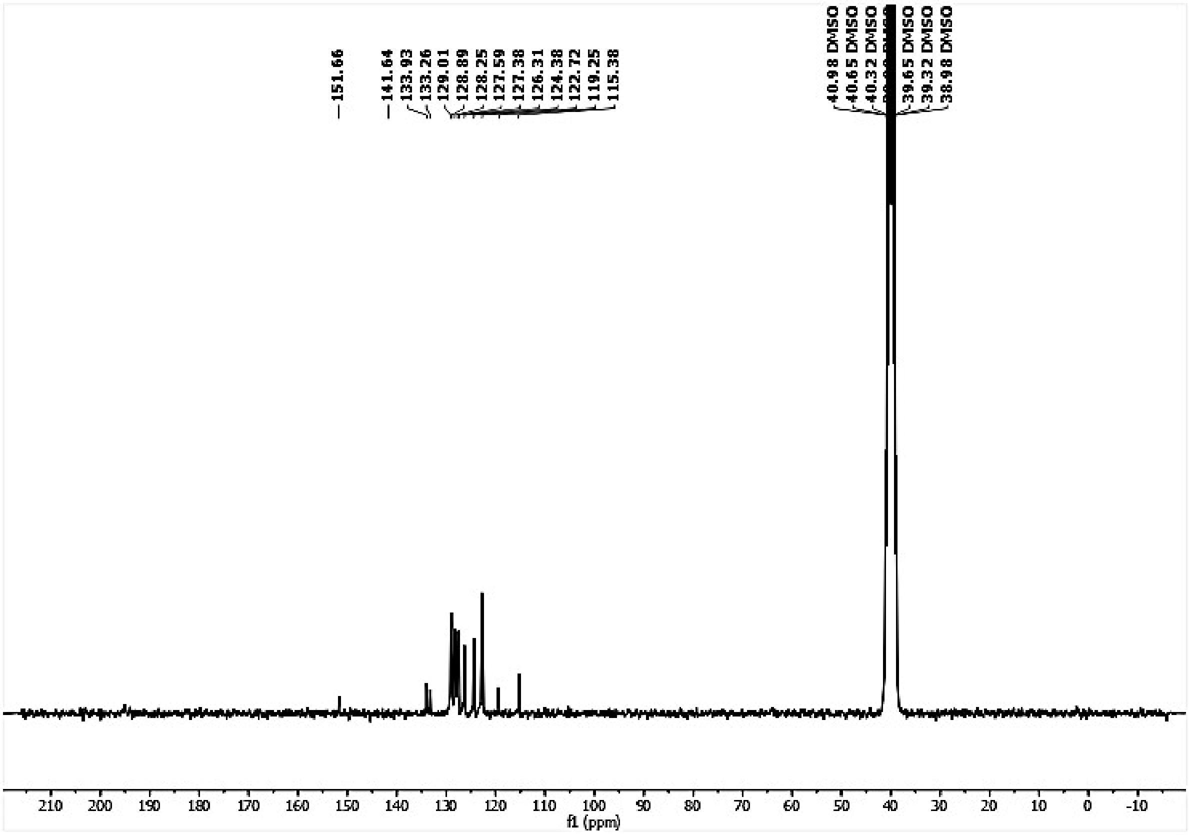

Figure S20. ^{13}CNMR spectra of 2-(6-bromonaphthalen-1-yl)-1*H*-benzo[*d*]imidazole (compound 3j).

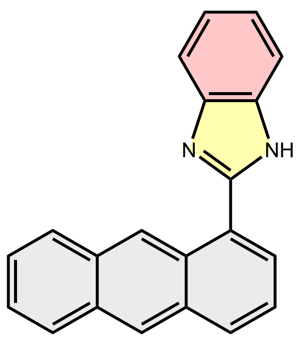

Compound 3h: 98% yield; ^{1}H NMR (400 MHz, DMSO-d_6) δ 13.08 (s, 1H), 8.86 (s, 1H), 8.23 (d, J = 8.2 Hz, 2H), 7.82 (s, 1H), 7.69 (d, J = 8.7 Hz, 1H), 7.62 (d, J = 6.9 Hz, 1H), 7.59 (d, J = 4.0 Hz, 2H), 7.59 – 7.44 (m, 3H), 7.33 (t, J = 5.7 Hz, 1H). ^{13}C NMR (101 MHz, DMSO) δ 149.99, 140.28, 135.11, 131.12, 131.02, 129.31, 128.99, 127.34, 126.31, 126.15, 126.08, 123.03, 122.07, 119.57, 111.91.

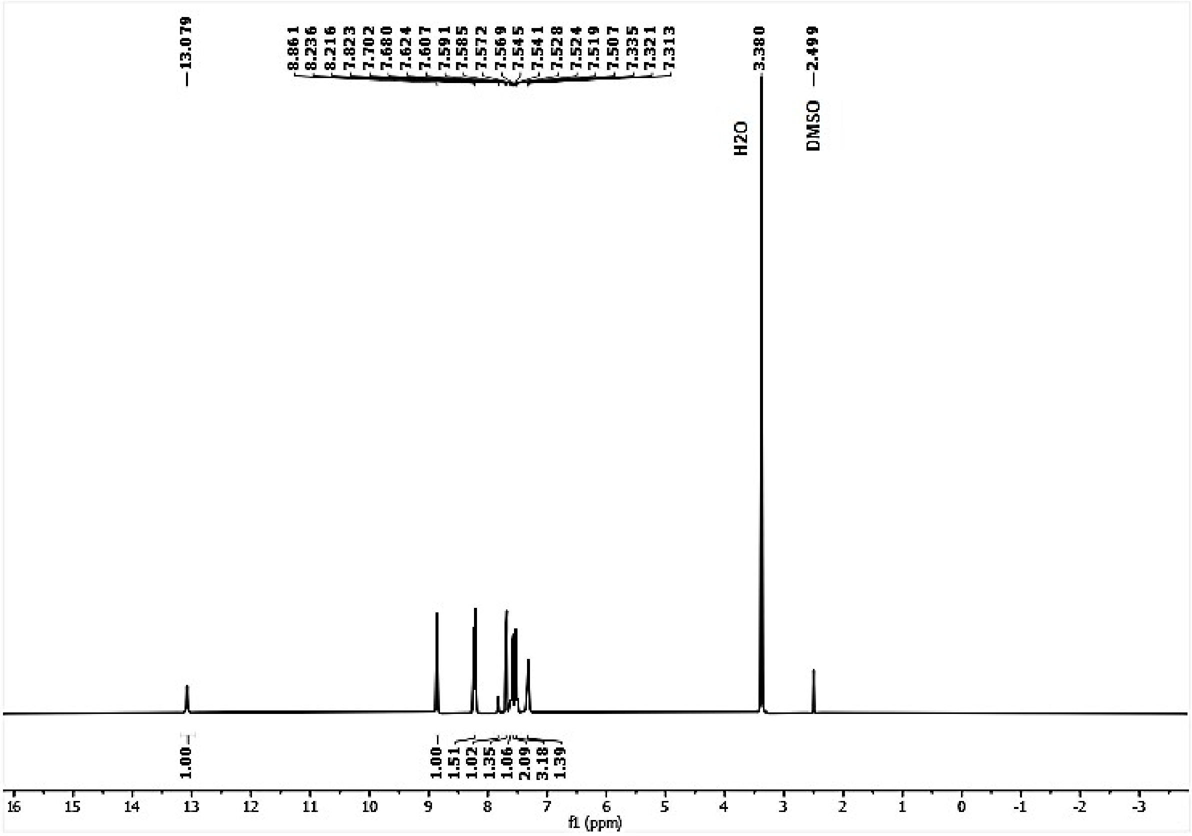

Figure S21. ^{1}HNMR spectra of 2-(anthracen-1-yl)-1*H*-benzo[*d*]imidazole (compound 3h).

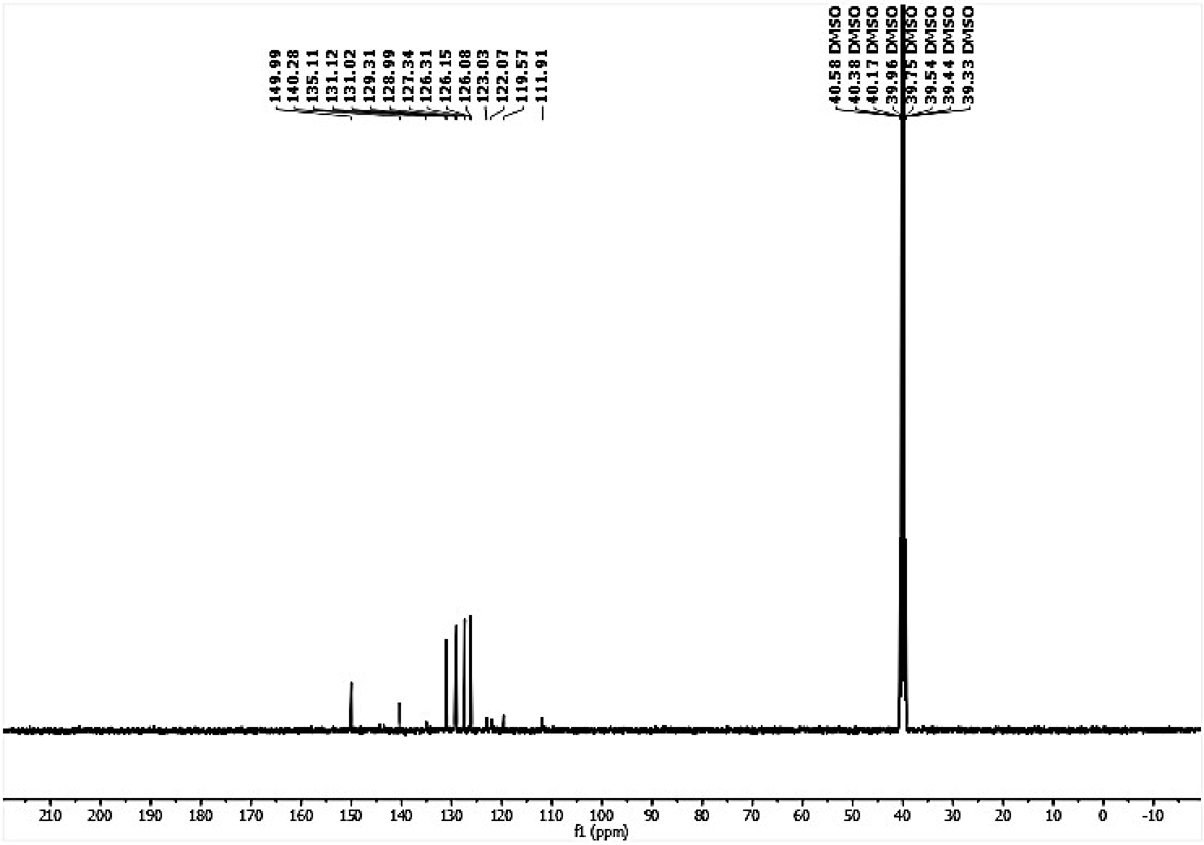

Figure S22. ^{13}CNMR spectra of 2-(anthracen-1-yl)-1*H*-benzo[*d*]imidazole (compound 3h).

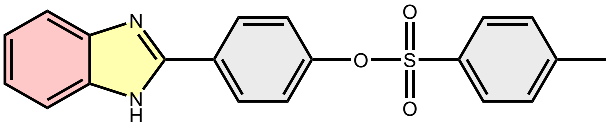

Compound 3i: 93% yield; ^{1}H NMR (250 MHz, DMSO-d_6) δ 12.52 (s, 1H), 8.14 (d, J = 7.9 Hz, 2H), 7.77 (d, J = 8.0 Hz, 2H), 7.59 (d, J = 5.2 Hz, 2H), 7.47 (d, J = 7.6 Hz, 2H), 7.23 – 7.20 (m, 4H), 2.41 (s, 3H). ^{13}C NMR (63 MHz, DMSO) δ 150.38, 146.43, 139.53, 131.74, 130.73, 129.50, 128.76, 128.60, 123.16, 122.89, 115.67, 115.29, 21.62.

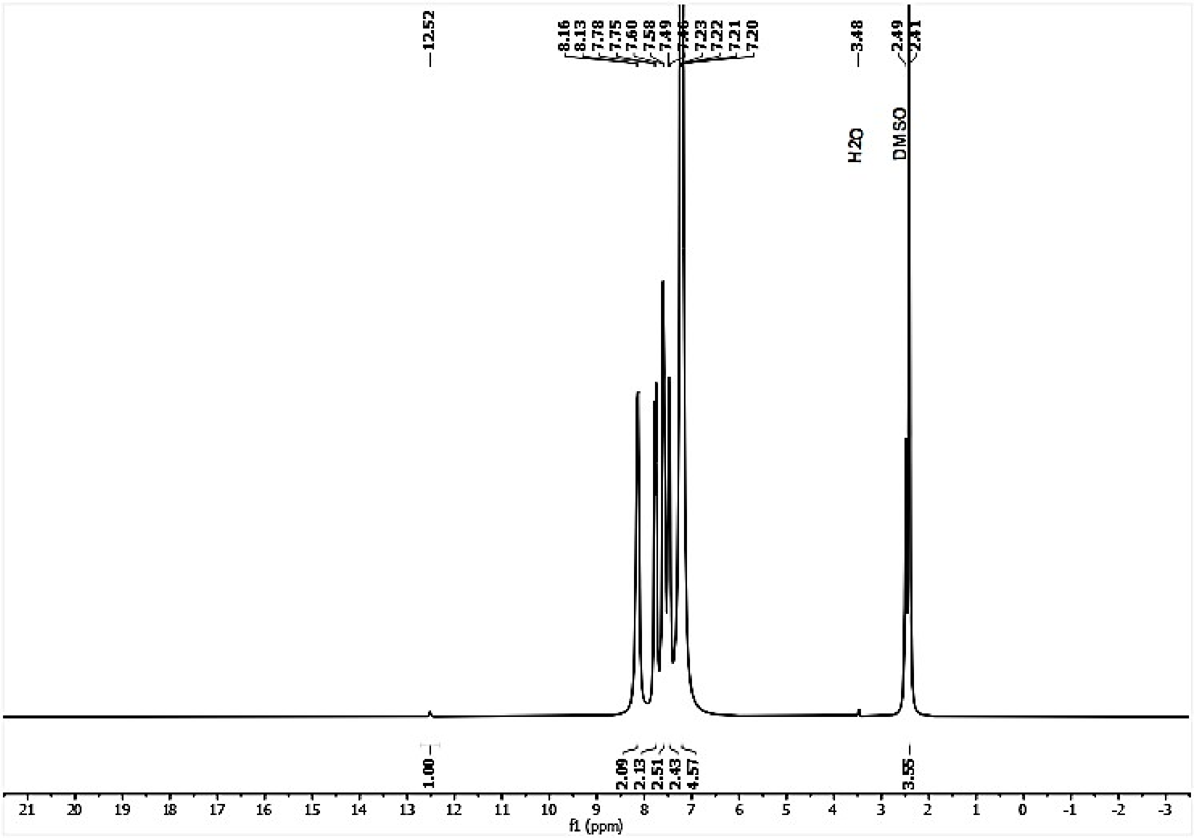

Figure S23. ^{1}HNMR spectra of 4-(1*H*-benzo[*d*]imidazol-2-yl)phenyl 4-methylbenzenesulfonate (compound 3i).

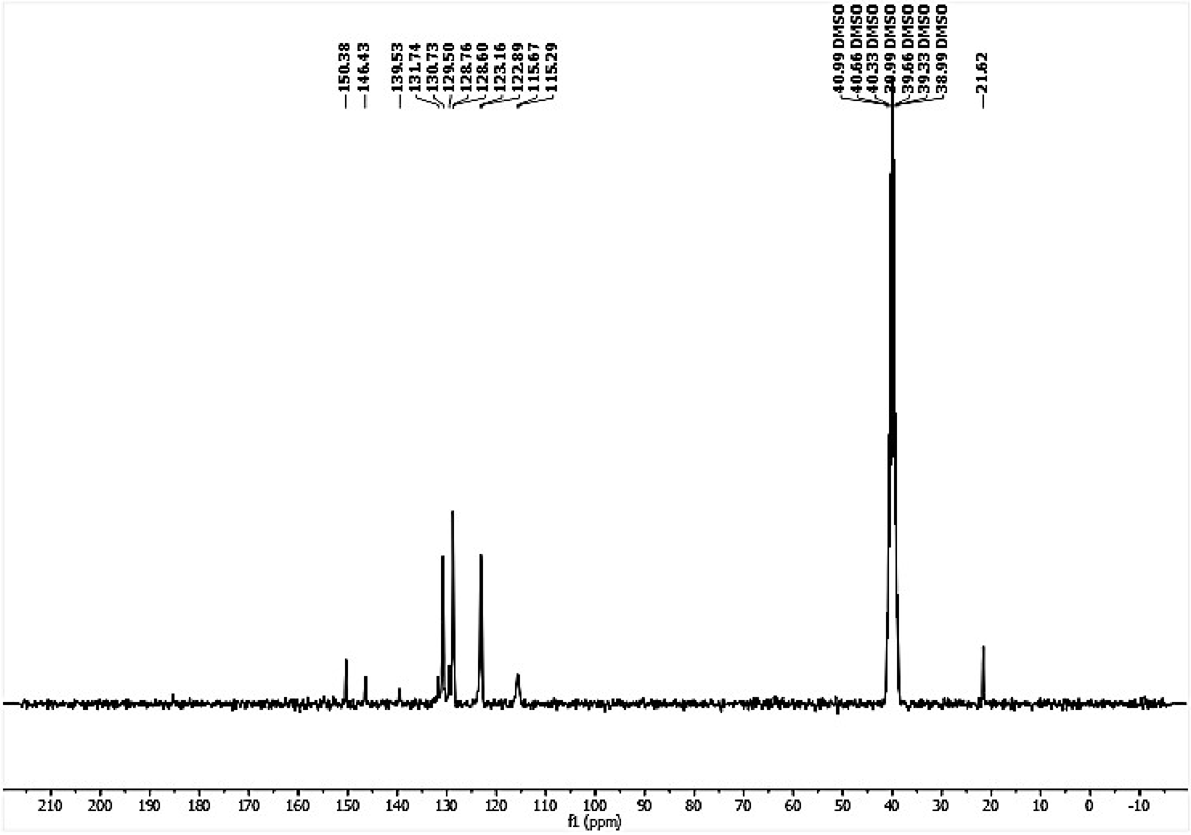

Figure S24. ^{13}CNMR spectra of 4-(1*H*-benzo[*d*]imidazol-2-yl)phenyl 4-methylbenzenesulfonate (compound 3i).
